# SHP2 deneddylation mediates tumor immunosuppression in colon cancer via the CD47/SIRP**α** axis

**DOI:** 10.1172/JCI162870

**Published:** 2023-02-15

**Authors:** Yiqing Li, Hui Zhou, Pan Liu, Dandan Lv, Yichun Shi, Bufu Tang, Jiaqi Xu, Tingting Zhong, Wangting Xu, Jie Zhang, Jianying Zhou, Kejing Ying, Yongchao Zhao, Yi Sun, Zhinong Jiang, Hongqiang Cheng, Xue Zhang, Yuehai Ke

**Affiliations:** 1 Department of Pathology and Pathophysiology, and Department of Respiratory Medicine at Sir Run-Run Shaw Hospital, Zhejiang University School of Medicine, Hangzhou, China.; 2 Zhejiang Cancer Hospital, Institute of Basic Medicine and Cancer (IBMC), Chinese Academy of Sciences, Hangzhou, China.; 3 Department of Respiratory Medicine at Sir Run-Run Shaw Hospital, Zhejiang University School of Medicine, Hangzhou, China.; 4 Key Laboratory of Imaging Diagnosis and Minimally Invasive Intervention Research at The Lishui Hospital, Zhejiang University School of Medicine, Lishui, China.; 5 Department of Pathology at Sir Run-Run Shaw Hospital, Zhejiang University School of Medicine, Hangou, China.; 6 Department of Respiratory Medicine at The First Affiliated Hospital, Zhejiang University School of Medicine, Hangzhou, China.; 7 Department of Urology at Sir Run-Run Shaw Hospital, Zhejiang University School of Medicine, Hangzhou, China.; 8 Cancer Institute of The Second Affiliated Hospital, and Institute of Translational Medicine, Zhejiang University School of Medicine, Hangzhou, China.; 9 Department of Pathology and Pathophysiology, and Department of Cardiology at Sir Run-Run Shaw Hospital, Zhejiang University School of Medicine, Hangzhou, China.

**Keywords:** Cell Biology, Immunotherapy, Macrophages, Signal transduction

## Abstract

SIPR*α* on macrophages binds with CD47 to resist proengulfment signals, but how the downstream signal of SIPR*α* controls tumor-infiltrating macrophages (TIMs) is still poorly clarified. Here, we report that the CD47/signal regulatory protein *α* (SIRP*α*) axis requires the deneddylation of tyrosine phosphatase SHP2. Mechanistically, Src homology region 2–containing protein tyrosine phosphatase 2 (SHP2) was constitutively neddylated on K358 and K364 sites; thus, its autoinhibited conformation was maintained. In response to CD47-liganded SIRP*α*, SHP2 was deneddylated by sentrin-specific protease 8 (SENP8), which led to the dephosphorylation of relevant substrates at the phagocytic cup and subsequent inhibition of macrophage phagocytosis. Furthermore, neddylation inactivated myeloid-SHP2 and greatly boosted the efficacy of colorectal cancer (CRC) immunotherapy. Importantly, we observed that supplementation with SHP2 allosteric inhibitors sensitized immune treatment–resistant CRC to immunotherapy. Our results emphasize that the CRC subtype that is unresponsive to immunotherapy relies on SIRP*α*^hi^SHP2^hi^NEDD8^lo^ TIMs and highlight the need to further explore the strategy of SHP2 targeting in CRC therapy.

## Graphical abstract

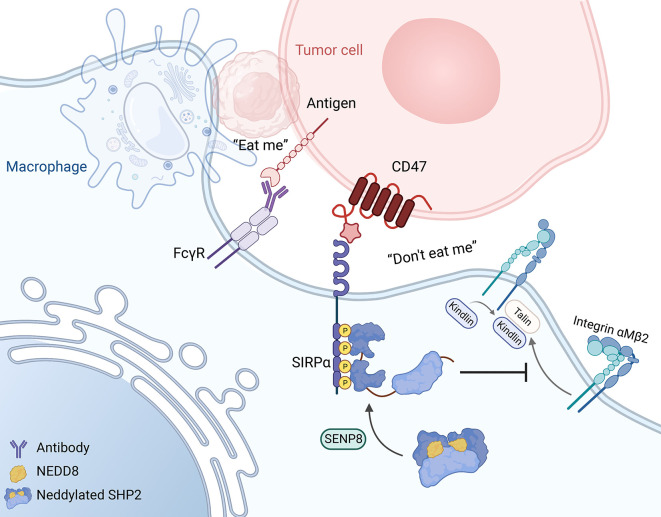

## Introduction

Reprogramming the immunosuppressive tumor microenvironment is vital in optimal tumor immunotherapy. Immune checkpoint inhibitors that target tumor-infiltrating lymphocytes have brought unprecedented success in the clinical efficacy of malignant tumor therapy (1, 2). However, the tumor microenvironment depends on a combination of both innate and adaptive immune cells, and except for infiltrated T cells, macrophages with protumor effects also have a critical impact on the outcome of immunotherapy (3, 4). Thus, harnessing tumor-infiltrating macrophages (TIMs) to reinvigorate immunosurveillance is a promising therapeutic strategy (5).

Macrophages contribute significantly to immune surveillance by engulfing tumor cells (6, 7). With the engagement of Fcγ receptors, macrophages also contribute to the clinical success of therapeutic antibodies by antibody-dependent cellular phagocytosis (ADCP) (8–10). Indeed, phagocytosis relies on a balance between pro- and antiengulfment signals of target cells. Initially observed on erythrocytes, CD47 inhibits phagocytosis by binding to its receptor, signal regulatory protein α (SIRPα), on circulating phagocytes (11). Both solid and hematologic malignancies overexpress CD47 to hijack the intrinsic mechanism and thereby protect cancer against the detection and clearance of the immune system (12, 13). To date, mounting studies have confirmed that blockade of CD47/SIRPα could reactivate the phagocytic efficacy of macrophages. In the mouse tumor model, administering tumor-opsonizing antibodies with inhibition of this phagocytosis checkpoint promoted therapeutic efficacy (14, 15), and SIRPα-deficient macrophages showed activation of cytotoxic T cells by conducting immunogenic antigen presentation (16). In particular, recent clinical studies have demonstrated that CD47/SIRPα axis disruption exhibits remarkable synergy and improved outcomes with therapeutic antibodies (17).

Mechanically, CD47-recognized SIRPα triggers phosphorylation of its immunoreceptor tyrosine-based inhibitory motif (ITIM) in the cytoplasmic region, which results in recruitment of the tyrosine phosphatases Src homology region 2-containing protein tyrosine phosphatase 1 (SHP1) and SHP2 for signal transduction (18, 19). Recent works have clarified that integrin is a downstream effector of SIRPα for cytoskeletal rearrangement, which is necessary for phagocytosis (20). Distinct paradigms of posttranslational modifications have also been identified as novel regulatory mechanisms of the CD47/SIRPα axis. Glutaminyl cyclase mediates N-terminal pyroglutamate of CD47 protein, which is considered to be vital for SIRPα binding (21). Macrophage SIRPα modulates phagocytic cup formation via a dephosphorylation cascade involving Y277 and Y1805 in myosin (22). These findings raise questions about the involvement of other posttranslational modifications in regulating the downstream signal of SIRPα.

Defined as ubiquitin-like modification (23), neddylation conducts the cycle of NEDD8 conjugation and deconjugation to the substrate. C-terminal glycine residue of NEDD8 is covalently attached to the ε-amino group of lysine on the target protein through an isopeptide bond. The neddylation system is composed of NEDD8-activating enzyme E1(heterologous dimer NAE1 and UBA3), NEDD8-conjugating enzyme E2 (UBE2M and UBE2F), E3 ligases, and NEDD8-specific proteases (including sentrin-specific protease 8 [SENP8] and JAB1/CSN5) (24). Although neddylation activation of Cullin-RING ubiquitin ligases for tumor cell survival is well established, increased non-Cullin substrates have been proposed as performing a wide variety of functions, in which the deconjugation cycle is specifically modulated by deneddylase SENP8. Recently, cGAS (25) and Myd88 (26) have been identified as neddylation substrates in macrophages, which is strong evidence for the involvement of neddylation in the tumor immune microenvironment.

In this study, we discovered that the CD47/SIRPα signal triggered substrate deneddylation in colorectal TIMs, and we showed that NEDD8 conjugation of the K358 and K364 in SHP2 was essential for maintaining its autoinhibitory conformation. Thus, SHP2 requires deneddylation by SENP8 to ensure its activation and recruitment toward SIRPα and to suppress macrophage phagocytosis. Furthermore, the combination of PDL1 therapy with SHP2 allosteric inhibitor sensitized immune treatment–resistant colorectal cancer (CRC) to immunotherapy. Our study provides insightful clues about the promising clinical benefits of the CD47/SIRPα axis blockade by SHP2 inhibition in the immune treatment of CRC.

## Results

### Neddylation in TIMs is crucial for CD47/SIPRα checkpoint inhibition.

**Figure 1:**
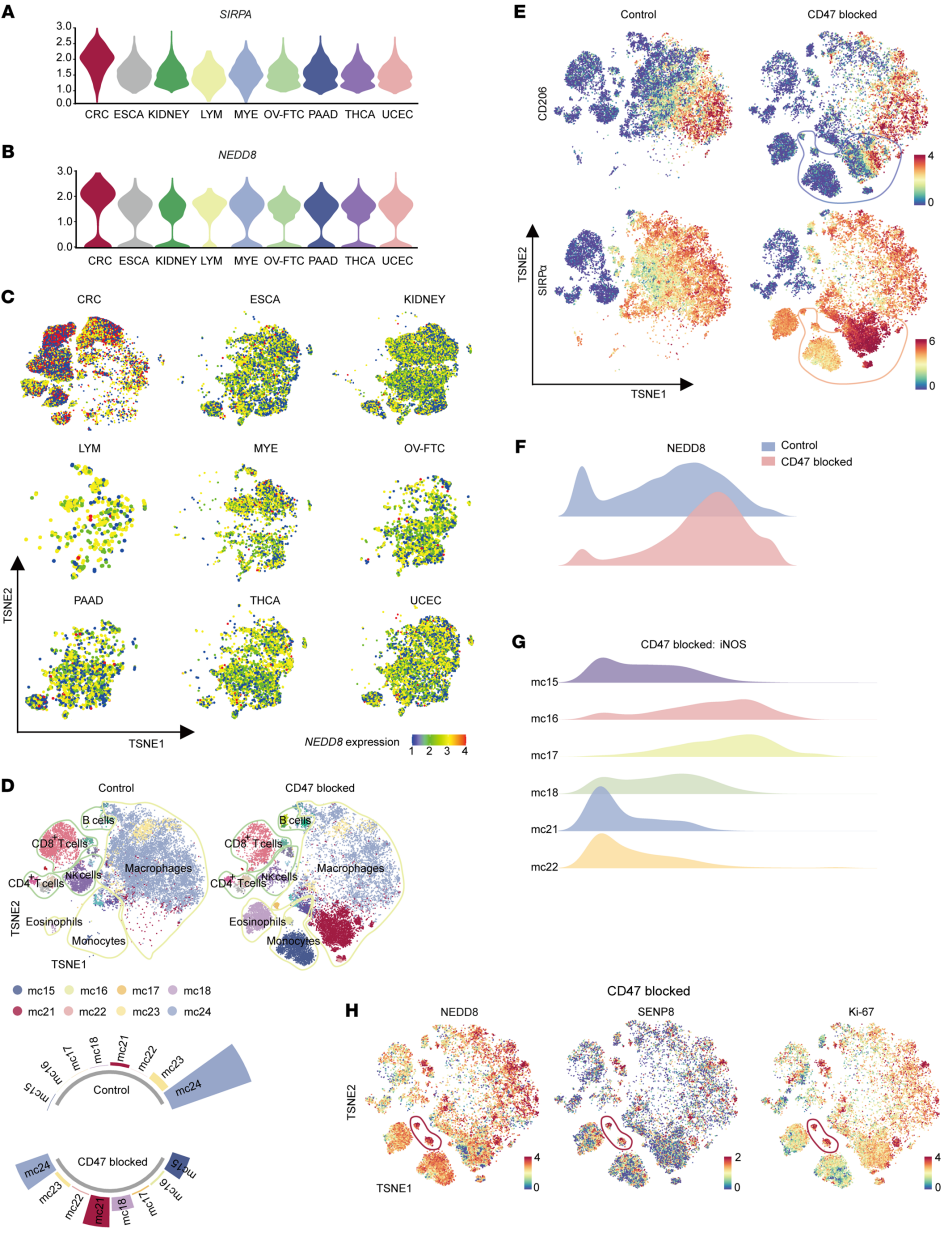
Neddylation and SENP8 play a regulatory role in response to CD47 stimulation. (**A**) Violin plot of *SIRPA* gene expression levels in different TIMs. (**B**) Violin plot of *NEDD8* gene expression levels in SIRPα^+^ TIMs. (**C**) TSNE plots of *NEDD8* gene expression levels in tumor-infiltrating myeloid cells. ESCA, esophageal carcinoma; KIDNEY, kidney cancer; LYM, lymphoma; MYE, myeloma; OV-FTC, ovarian or fallopian tube carcinoma; PAAD, pancreatic adenocarcinoma; THCA, thyroid carcinoma; UCEC, uterine corpus endometrial carcinoma. (**D**) TSNE plot shows lineages of tumor-infiltrating leukocytes in control (left) and CD47/SIRPα–blocking peptide–treated (Pep-20, 2 mg/kg, right) MC38 s.c. tumors (*n* = 6). mc numbers refer to major varied mouse clusters. (**E**) TSNE visualization of CD206 and SIRPα expression groups shown in **D**. (**F**) NEDD8 expression in total tumor-infiltrating monocytes/macrophages of groups show in **D**. (**G**) iNOS expression of major varied mouse clusters of Pep-20–treated groups. (**H**) TSNE visualization of NEDD8, SENP8, and Ki-67 expression in Pep-20–treated groups; mc16 and mc17 are circled.

First, we analyzed the TIMs that are derived from 9 human cancer types by using published scRNA-Seq data (27, 28). TIMs of CRC showed the highest expression levels of *SIRPA* (Figure 1A). *CD47*, the ligand of SIRPα, was shown to be elevated in CRC tumors by RNA-Seq data from the colon adenocarcinoma (COAD) cohort of The Cancer Genome Atlas (TCGA) database (Supplemental Figure 1A; supplemental material available online with this article; https://doi.org/10.1172/JCI162870DS1). Intriguingly, extracted SIRPα^+^ clusters of TIMs specifically possessed a predominant expression level of *NEDD8* in CRC (Figure 1B). Likewise, our data reported a distinctive *NEDD8* expression pattern of tumor-infiltrating myeloid cells in CRC (Figure 1C).

Therefore, we hypothesized that neddylation affected the CD47/SIRPα axis in TIMs. To test this hypothesis, pep-20, a CD47/SIRPα interaction-blocking peptide (29), was employed for the treatment of the s.c. MC38 colon carcinoma model of C57BL/6 mice. Then tumors were digested to conduct cytometry by time of flight (CyTOF) analyses of CD45^+^ cells after drug administration. As a result of the large loss of macrophage clusters (mc23, mc24) from the total pool of immune cells, we observed an increase in the abundance of monocytes/macrophages (mc15, mc16, mc17, mc18, mc21, mc22) in the CD47-blocked group (Figure 1D and Supplemental Figure 1B). With decreased M2 marker CD206, the emerging SIRPα^+^ monocytes/macrophages demonstrated stronger antitumor activity compared with original macrophage populations (Figure 1E). Indeed, NEDD8 expression was upregulated by CD47/SIPRα axis blockage in total monocytes/macrophages (Figure 1F). More importantly, among the emerging SIRPα^+^ monocyte/macrophage populations of the CD47-blocked group, the mc16 and mc17 clusters, which were a group of NEDD8^hi^SENP8^hi^Ki-67^hi^ clusters, exhibited a prevailing expression of M1 marker iNOS (Figure 1, G and H). These results indicate that neddylation may be involved in SIRPα signaling in TIMs of CRC.

### SENP8 regulates deneddylation in TIMs to respond to tumor cell CD47.

**Figure 2:**
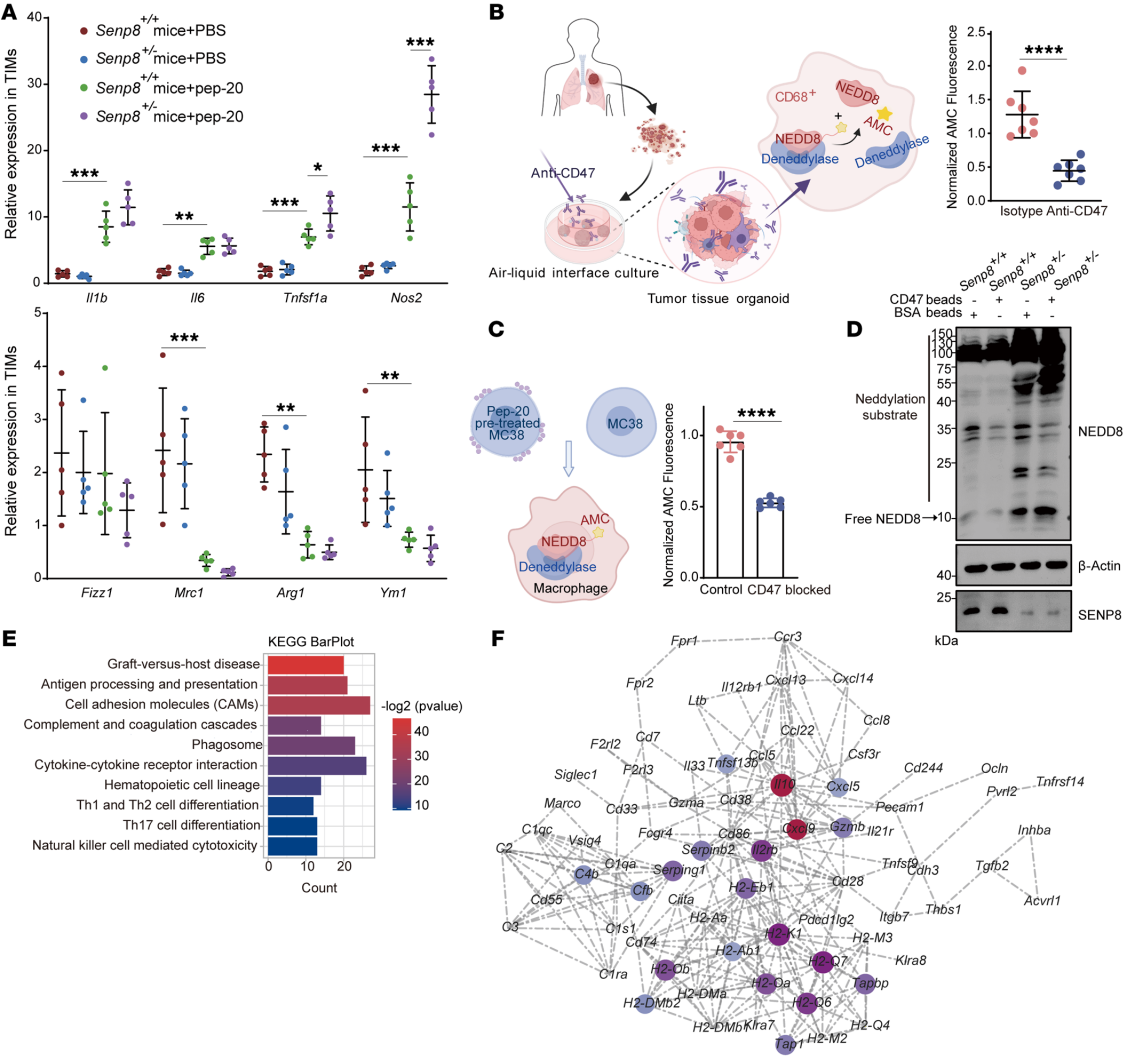
Phenotypes of TIMs are altered by SENP8 in CD47/SIPRα signaling. (**A**) RNA expression of phenotype markers in TIMs from indicated groups (*n* = 5). (**B**) Declined deneddylation levels of CD68^+^ macrophages in tumor tissue organoids under CD47 blockage (*n* = 7). (**C**) Declined deneddylation levels of BMDMs under CD47 blockage (*n* = 6). (**D**) Western blot of neddylation substrates from indicated groups. (**E**) KEGG analysis classifying genes into biological process groups. (**F**) Cytoscape network for classified genes imputed from KEGG analysis. Data are represented as mean ± SD. *P < 0.05; **P < 0.01; ***P < 0.001; ****P < 0.0001. Two-tailed, unpaired Student’s *t* test (**B** and **C**); 2-way ANOVA followed by Tukey’s post hoc test (**A**).

Because SENP8 is the major deneddylase to deconjugate NEDD8 from the substrates (30), the *Senp8* heterozygous mouse strain was generated (Supplemental Figure 1C). Specifically, TIMs lacking SENP8 acquired more proinflammatory features under CD47 blockage (Figure 2A). Dynamic and reversible neddylation relies on the isopeptide bond between NEDD8 and substrates. Thus, NEDD8-AMC (31), whose isopeptide hydrolysis results in the release of the intensive fluorescence of AMC, was utilized to detect deneddylation. We cultured tumor microenvironment–preserved organoids (32) derived from surgical tumor resections of humans and treated them with anti-CD47 antibodies or isotype control (Supplemental Figure 1D). Extracted CD68^+^ macrophages from organoids had lower isopeptidase activity on NEDD8-AMC when the CD47 checkpoint was inhibited (Figure 2B). Similarly, murine bone marrow–derived macrophages (BMDMs) were cocultured with MC38 cells, and blocking the CD47/SIRPα axis resulted in descending deneddylation of BMDMs (Figure 2C).

Incubation of *Senp8^+/+^* and *Senp8^+/–^* BMDMs with CD47-coated polystyrene triggered a reshaped neddylation profile of substrates. Western blotting demonstrated that SENP8 deletion boosted neddylation of endogenous substrates in lane 3 and that SENP8 deletion abolished substrates’ deneddylation in response to CD47 in lane 4, particularly for proteins with molecular weights larger than 40 kDa (Figure 2D). These results indicate SENP8-regulated deneddylation participates downstream of SIRPα signaling in TIMs of CRC.

In addition, RNA-Seq was performed to show the transcriptional differences between CD47-treated *Senp8^+/+^* and *Senp8^+/–^* BMDMs (Supplemental Figure 1E). The presence of KEGG term cell adhesion molecules and phagosome represented a leading role that neddylation played in SIPRα-mediated phagocytosis (Figure 2E). Furthermore, regulatory network analysis of KEGG results using Cytoscape (https://cytoscape.org/) highlighted *Il10* and *Cxcl9*, which revealed neddylation-regulated phenotype switching and chemotactic responses of macrophages (Figure 2F).

### Tyrosine phosphatase SHP2 is covalently modified by NEDD8 on K358 and K364 sites.

**Figure 3:**
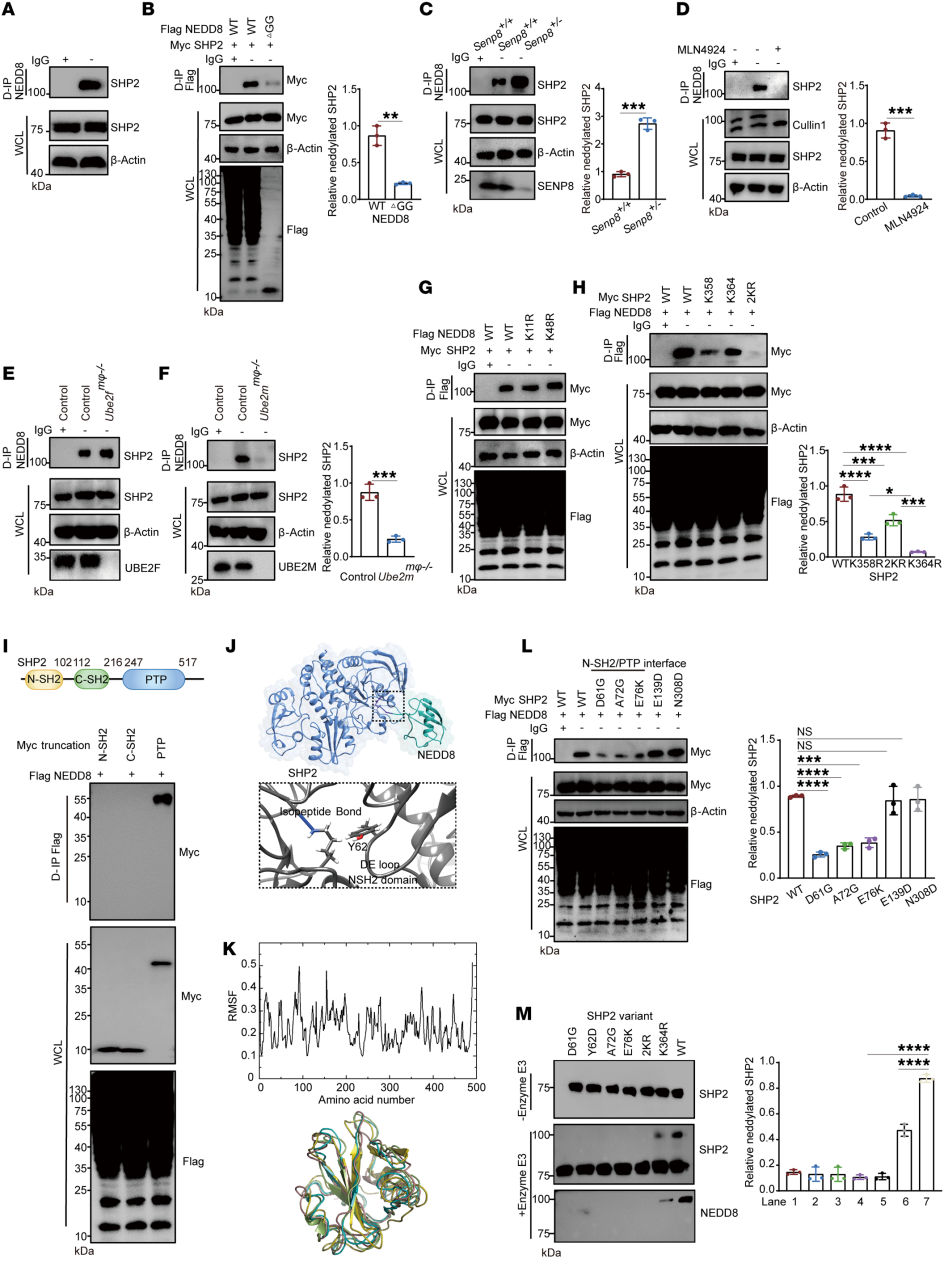
SHP2 is a genuine substrate of NEDD8. (**A**) Western blot indicating SHP2 neddylation of BMDMs. (**B**) Western blot indicating NEDD8-SHP2 conjugation relied on the isopeptide bond of HEK293T cells (*n* = 3). (**C**) Western blot indicating depletion of SENP8 enhanced SHP2 neddylation of BMDMs (*n* = 3). (**D**–**F**) SHP2 neddylation in BMDMs was attenuated by E1 inhibitor MLN4924 (1 μM, 8 hours) (**D**) or deletion of UBE2M (**F**) but not UBE2F (**E**) (*n* = 3). (**G**) Western blot indicating Poly-NEDD8 chain was not involved in SHP2 neddylation of HEK293T cells (*n* = 3). (**H**) Western blot indicating sites of SHP2 neddylation of HEK293T cells (*n* = 3). (**I**) Western blot indicating domains of SHP2 neddylation of HEK293T cells (*n* = 3). (**J**) Docking of molecules between NEDD8 and SHP2. (**K**) Upper panel: RMSF of SHP2 residents in MD. Lower panel: color overlay of the time-frame configurations in 1–100 amino acid area of SHP2. (**L**) Western blot indicating the state of SHP2 variant neddylation of HEK293T cells (*n* = 3). (**M**) Western blot indicating in vitro neddylation assay (*n* = 3). Recombinant SHP2 and its indicated mutation (1 μM), E3 XIAP (2.5 μM). D-IP, denaturing IP; WCL, whole-cell lysate. Data are represented as mean ± SD. *P < 0.05; **P < 0.01; ***P < 0.001; ****P < 0.0001; nonsignificant (NS), *P* > 0.05. Two-tailed, unpaired Student’s *t* test (**B**, **C**, **D**, **F**); 2-way ANOVA followed by Tukey’s post hoc test (**H**, **L**, **M**);

To investigate the potential neddylation substrates implicated in the CD47/SIRP axis, the in silico tool NeddyPreddy (33) was utilized. Interestingly, SHP2, the downstream target of SIRPα, was discovered to be a candidate for neddylation substrate (Supplemental Figure 2A). As mass analysis of SHP2 co-IP proteins presented the peptide belonging to NEDD8-conjugating E2 UBE2M (Supplemental Figure 2B), we performed denaturing IP to detect SHP2 neddylation. In particular, BMDMs reported a higher molecular-weight band induced by endogenous NEDD8 conjugation with SHP2, but not its homologous protein SHP1 (Figure 3A and Supplemental Figure 2C). The NEDD8/SHP2 conjugation was abolished by NEDD8-ΔGG truncation, which lacked specific residues to covalently attach with its substrates (Figure 3B). Moreover, we confirmed that SENP8 is the specific deneddylase for SHP2 by silencing or overexpressing experiments in HEK293T cells (Supplemental Figure 2, D and E), and elevated SHP2 neddylation was also found in *Senp8^+/–^* BMDMs (Figure 3C). To confirm the NEDD8-activating E1 complex of SHP2 neddylation cascade, BMDMs were treated with MLN4924 (34), a specific inhibitor of NEDD8-activating E1 that blocks neddylation, and the neddylated band of SHP2 was eliminated under MLN4924 treatment (Figure 3D). To investigate the NEDD8-conjugating E2 of the SHP2 neddylation cascade, macrophage-specific *Ube2f^mφ–/–^* and *Ube2m^mφ–/–^* mice were generated, and endogenous neddylated SHP2 was found to decrease only in *Ube2m^mφ–/–^* BMDMs (Figure 3, E and F, and Supplemental Figure 2F). Similarly, an upregulated SHP2 neddylation was achieved by ectopically expressing UBE2M but not UBE2F in HEK293T cells (Supplemental Figure 2G). These observations suggest that UBE2M is the specific E2 for SHP2.

SHP2 neddylation produced a shift larger than 8 kDa, which is the molecular weight of mono-NEDD8 protein. To determine whether SHP2 neddylation involves the poly-NEDD8 chain, NEDD8 mutation (K11R/K48R) (35) was induced to limit chain formation. The unaltered SHP2 neddylation proved that the poly-NEDD8 chain was not involved in NEDD8/SHP2 conjugation (Figure 3G). Given the predicted mutation K358R from the in silico tool NeddyPreddy did not entirely eliminate SHP2 neddylation (Supplemental Figure 2H), we assumed that multiple lysine residues were modified by mono-NEDD8 in SHP2. Lysines located near each site have been reported to be redundant neddylation sites for certain substrates (36). K364 is an evolutionarily conserved site adjacent to K358, and the combined mutation K358R/K364R (referred to as 2KR) displayed a marked reduction in SHP2 neddylation (Figure 3H). Indeed, The K358 and K364 residues are accessible on the surface of SHP2, as they belong to the same loop between βE and βF of the catalytic protein tyrosine phosphatase (PTP) domain (37). We concluded that K358 and K364 were the 2 main neddylated sites of SHP2 because the neddylated band of the PTP domain was about 15 kDa larger than the unmodified PTP domain (Figure 3I). Moreover, the aligned sites of K358 and K364 in homologous family protein SHP1 were arginines that do not allow neddylation (Supplemental Figure 2I).

### NEDD8 conjugation impairs SHP2 activation.

A crystal structure of the K364-neddylated SHP2 provides a basis for understanding how neddylation regulates SHP2 function via conformational changes. Encoded by *PTPN11*, SHP2 consists of 2 SH2 domains as well as a central PTP domain. SHP2 adopts a closed conformation engaged by the SH2 domain and the PTP domain in the basal state, and SH2 domains binding with tyrosine-phosphorylated ligands lead the autoinhibition into an open state for dephosphorylation (37). The SHP2 K364 isopeptide bond was located near the DE loop and hidden in the N-SH2 and PTP domain interface (Figure 3J). To understand the nature of the allosteric network that is influenced by neddylation, we performed molecular dynamics (MD) simulations of neddylated SHP2 and bisphosphorylated IRS-1 peptide (38) (2P-IRS-1), which stimulated SHP2 phosphatase activity. MD trajectory analysis revealed that the N-SH2 and the C-SH2 domains have higher atomic root mean square fluctuations (RMSF), which represent the flexibility and motion intensity of protein amino acids (Figure 3K).

**Figure 4:**
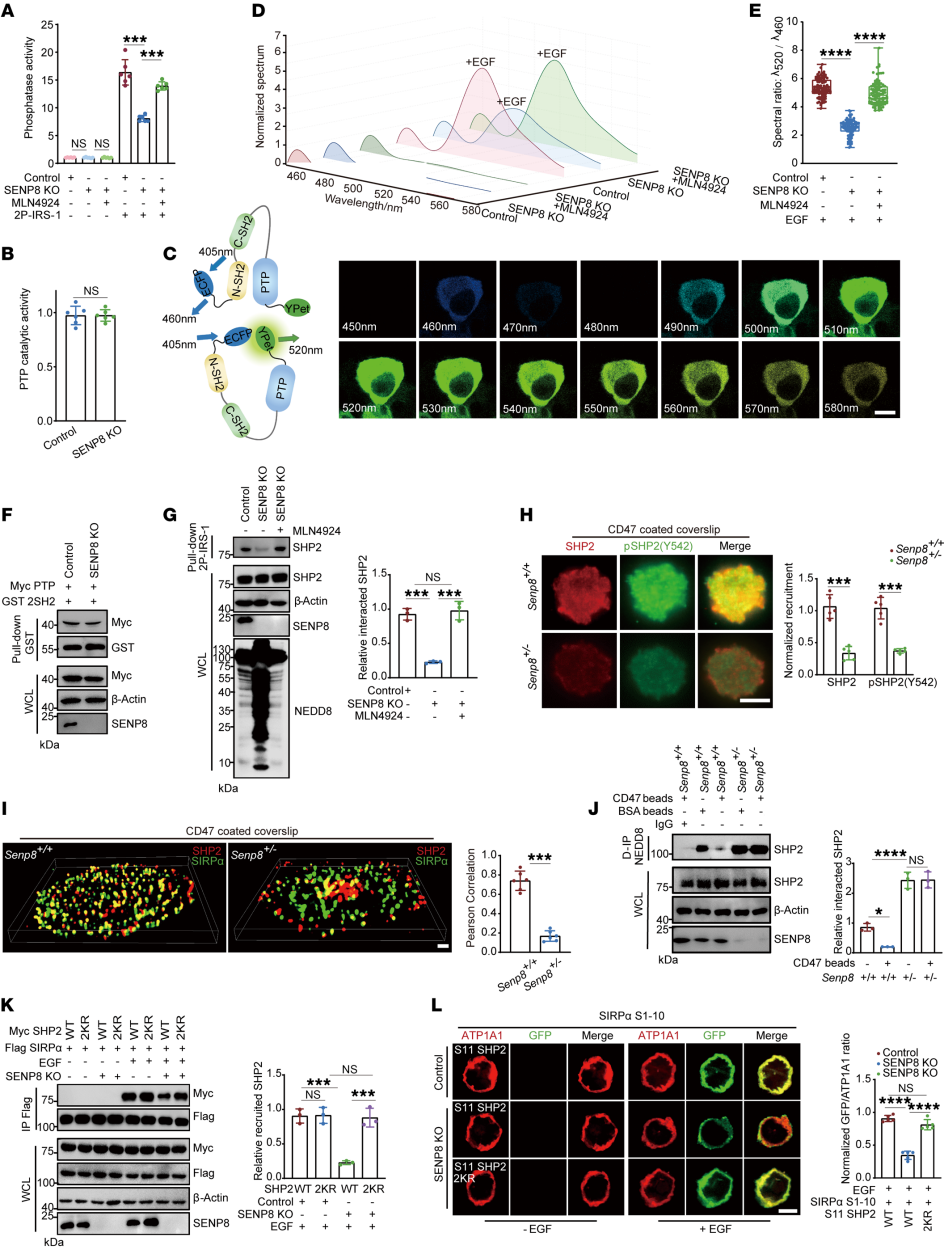
SENP8 deconjugates NEDD8 from SHP2 to facilitate its recruitment toward SIPRα. (**A**) Phosphatase activity was detected for IP of HEK293T cells (*n* = 6). 2P-IRS-1 incubation was conducted in vitro (1 mM, 30 minutes). (**B**) Catalytic activity of PTP was detected for IP of HEK293T cells (*n* = 6). (**C**) Spectral images of detected emission of SHP2 biosensor under EGF stimulation (10 ng/ml, 15 minutes). Scale bar: 10 μm. Numbers in images show wavelengths. (**D**) Normalized intensity of emitted fluorescence spectrum shown in **C**. (**E**) The spectral ratio revealed in **C** (cell number = 100). (**F**) Western blot indicating affinity of 2SH2 domain and PTP domain in HEK293T cell lysates. (**G**) Western blot indicating affinity of 2P-IRS-1 and SHP2 in HEK293T cell lysates (*n* = 3). (**H**) Representative TIRF fluorescent images of BMDMs (*n* = 5). Scale bar: 10 μm. (**I**) TIRF-STORM images of SIRPα and SHP2 colocalization in BMDMs (*n* = 6). Scale bar: 1 μm. (**J**) Western blot indicating SHP2 neddylation of BMDMs under CD47 stimulation (*n* = 3). (**K**) Western blot indicating SHP2 recruitment by the SIRPα receptor was affected by neddylation under EGF stimulation (10 ng/ml, 15 minutes) in HEK293T cells (*n* = 3). (**L**) Confocal microscopy visualization of the recruitment of SHP2 toward the SIRPα receptor under EGF stimulation (10 ng/ml, 15 minutes). Scale bar: 10 μm. *n* = 5. Data are represented as mean ± SD. *P < 0.05; ***P < 0.001; ****P < 0.0001; NS, *P* > 0.05. Two-tailed, unpaired Student’s *t* test (**B**); 1-way ANOVA followed by Tukey’s post hoc test (**A**, **E**, **G**, **J**, **K**, **L**); 2-way ANOVA by Tukey’s post hoc test (**H**); Pearson’s correlation (**I**).

For detecting the effect of NEDD8 conjugation on the N-SH2/PTP interface, we introduced disease-related SHP2 mutations that lead to a relatively open conformation. Only the mutations clustered at the N-SH2/PTP interface resulted in defective neddylation levels (Figure 3L). In order to confirm this phenomenon, we identified neddylation E3 for SHP2 and conducted in vitro neddylation assays using recombinant protein. XIAP was proved to decrease the exogenous SHP2 neddylation level effectively (Supplemental Figure 2, J and K). In addition, RBX1, a NEDD8 E3 ligase responsible for Cullin1 (39), had no significant effect on SHP2 neddylation (Supplemental Figure 2L). When XIAP was added to the reaction system, D61G, Y62D, and E76K mutations, which clustered at the N-SH2/PTP interface, showed decreased neddylation (Figure 3M). Furthermore, SHP2 of different neddylation levels was incubated with 2P-IRS-1. Under stimulation, neddylation dramatically attenuated the phosphatase activity of SHP2 (Figure 4A). Considering that NEDD8 conjugation did not influence the catalytic activity of its PTP domain (Figure 4B), we concluded that neddylation harmed SHP2 activation in a conformation-dependent way.

Then fluorescence resonance energy transfer (FRET) experiments (40) of the SHP2 biosensor were conducted to investigate its activation (Supplemental Figure 3A). Technically, as the N-terminal ECFP and C-terminal Ypet are separated in the basal state, 405 nm excitation generates 460 nm emission, whereas SHP2 conformational transition leads to 520 nm emission following EGF stimulation (Figure 4C). The spectrum of SHP2 biosensor emission showed that neddylation limited the ability of SHP2 to transition from closed to open conformation (Figure 4, D and E). Since the conformational transition of SHP2 is dependent on the competing affinity between the SH2 domain and the phosphorylated ligand or PTP domain, the corresponding affinities were investigated. Pull-down experiments revealed that neddylation of the PTP domain had no effect on its affinity with the SH2 domain (Figure 4F). However, neddylation inhibited SHP2 binding to tyrosine-phosphorylated ligands (Figure 4G). Furthermore, MST revealed a higher *K_D_* of the interaction between 2P-IRS-1 and NEDD8-SHP2 conjugation (Supplemental Figure 3B).

### SENP8-mediated deneddylation is required for SIRPα recruiting SHP2.

Given that neddylation prevented SHP2 from binding to activating ligands, we wondered how neddylation affected SHP2 in the CD47/SIRPα axis. Our data showed that the phosphorylated SIRPα ITIM motifs recruited and activated SHP2 (Supplemental Figure 4, A–C). Allosteric inhibitor SHP099 (41) for stabilizing SHP2 and phospho-ligand–binding deficient SH2 domain mutation (42) (2RA as R32A/R138A) both led to eliminating SHP2 binding with SIRPα (Supplemental Figure 4, D and E). These results indicate that neddylation might prevent SIRPα from recruiting SHP2.

Then, total internal reflection fluorescence (TIRF) microscopy, which has a selective spatial resolution of the plasma membrane region, was utilized to examine the interaction of SHP2 and SIRPα. When *Senp8^+/–^* BMDMs were cultured on a CD47-coated coverslip, successively recruited SHP2 and Y542 phosphorylated SHP2, which is an indicator of SHP2 activation, showed a significant decline (Figure 4H). Similarly, stochastic optical reconstruction microscopy (TIRF-STORM) imaging using superresolution validated abrogated colocalization of SIRPα and neddylated SHP2 (Figure 4I). In contrast, we did not detect an aberrant membrane location of neddylated SHP2 in the basal state (Supplemental Figure 4, F and G). Notably, incubation of BMDMs with CD47-coated beads decreased SHP2 neddylation, which was facilitated by SENP8 (Figure 4J). Thus, we concluded that NEDD8 was deconjugated from SHP2 by SENP8 in response to being recruited and activated by SIRPα.

Biochemically, the interaction between SHP2 and the introduced SIRPα receptor was rescued by 2KR mutations in SENP8-deficient HEK293T cells (Figure 4K). We also established the bimolecular fluorescence complementation (BiFC) system (43) by fusing complementary parts of GFP, GFP S1–10, to the C-terminus of SIRPα and GFP S11 to the N-terminus of SHP2. GFP signal was induced by the interaction between SIRPα and SHP2 on the plasma membrane. The SHP2 2KR mutation in SENP8-KO HEK293T cells totally restored the reduced GFP signal to the original level (Figure 4L). Moreover, a nonredundant role of SHP2 was found, as its homologous family protein SHP1 displayed unaffected binding with SIRPα under SENP8 disruption (Supplemental Figure 4H).

### SHP2 inactivates α_M_β_2_ integrin to suppress phagocytosis via the CD47/SIRPα axis.

**Figure 5:**
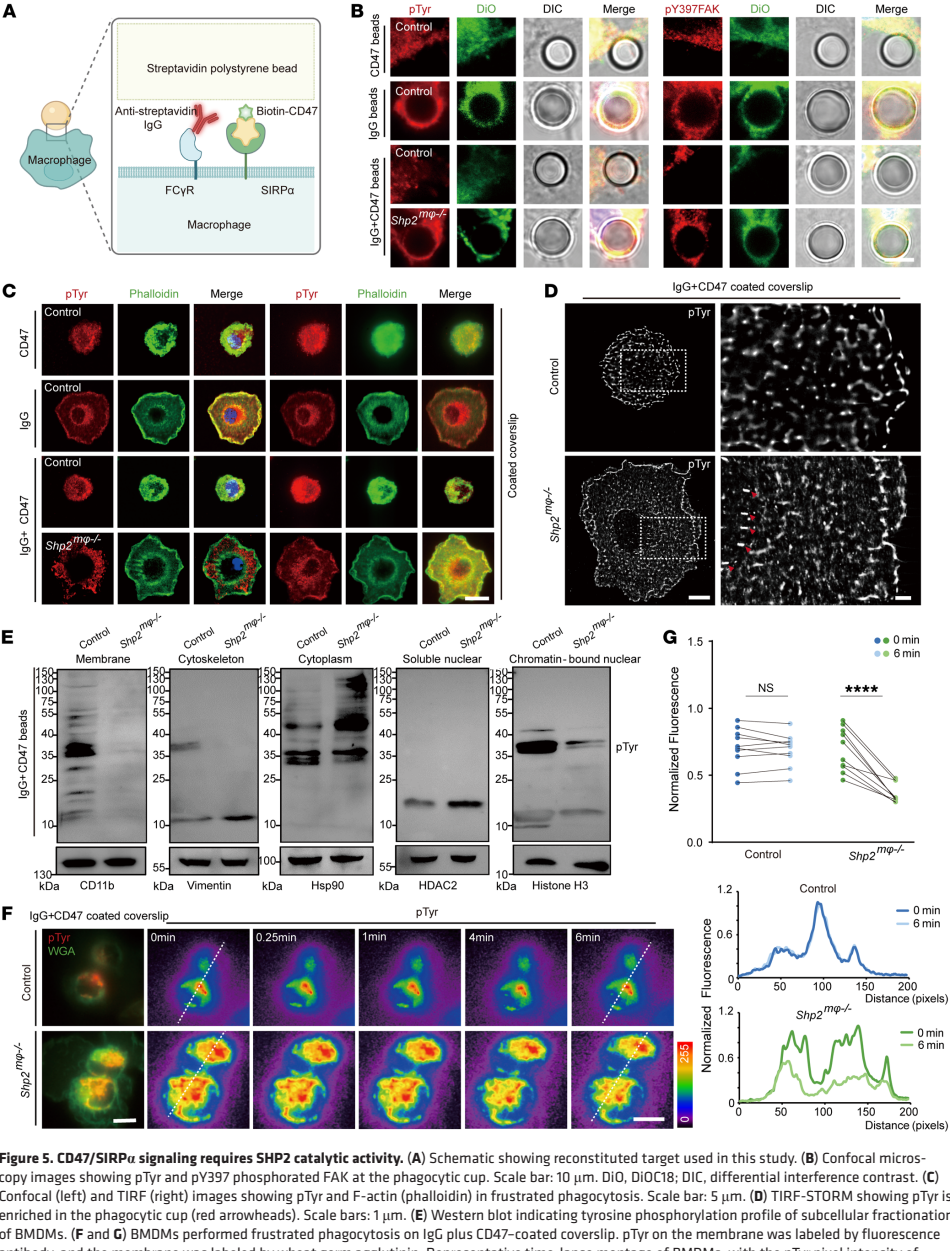
CD47/SIRPα signaling requires SHP2 catalytic activity. (**A**) Schematic showing reconstituted target used in this study. (**B**) Confocal microscopy images showing pTyr and pY397 phosphorated FAK at the phagocytic cup. Scale bar: 10 μm. DiO, DiOC18; DIC, differential interference contrast. (**C**) Confocal (left) and TIRF (right) images showing pTyr and F-actin (phalloidin) in frustrated phagocytosis. Scale bar: 5 μm. (**D**) TIRF-STORM showing pTyr is enriched in the phagocytic cup (red arrowheads). Scale bars: 1 μm. (**E**) Western blot indicating tyrosine phosphorylation profile of subcellular fractionation of BMDMs. (**F** and **G**) BMDMs performed frustrated phagocytosis on IgG plus CD47–coated coverslip. pTyr on the membrane was labeled by fluorescence antibody, and the membrane was labeled by wheat germ agglutinin. Representative time-lapse montage of BMDMs, with the pTyr pixel intensity of color-coded values indicated by the color wedge on the right. Scale bars: 5 μm (**F**). Graph of mean fluorescence intensity of pTyr over time during spreading (cell number = 10) (**G**). Data are represented as mean ± SD. ****P < 0.0001; NS, *P* > 0.05. Two-way ANOVA followed by Tukey’s post hoc test (**G**).

Macrophages mediate ADCP by recognizing and destroying antibody-opsonized cells in cancer therapy (44). As CD47/SIRPα engagement disrupts ADCP (45) and SHP2 serves as a signal molecule downstream of SIRPα, we wanted to investigate how SHP2 regulates the CD47 checkpoint. Therefore, reconstituted targets imitating antibody-opsonized tumor cells were used (Figure 5A), and macrophage-specific *Shp2^mφ–/–^* mice were generated (Supplemental Figure 5A). SHP2-deficient BMDMs showed restoration of the phagocytic cup outlined by tyrosine-phosphorylated proteins, including its substrate FAK (46), when challenged with coated beads (Figure 5B). For a more direct visualization of phagocytic cups, frustrated phagocytosis was conducted. Similarly, TIRF microscopy showed that ruffle-like pseudopods of the phagocytic cup in SHP2-deficient BMDMs were enriched with phospho-tyrosines (Figure 5C). In addition, the TIRF-STORM images of SHP2-deficient BMDMs depicted phospho-tyrosine relocation toward the phagocytic cup more clearly (Figure 5D). Considering that cytoskeletal rearrangement and membrane deformation are involved in phagocytosis, we further explored the spatially specific distribution of increased phosphorylation resulting from SHP2 deletion. Thus, a dynamic tyrosine phosphorylation profile was also depicted in different subcellular fractionations. Intriguingly, a decrease in phospho-tyrosines was exhibited by SHP2-deficient BMDMs in the membrane extract (Figure 5E). To validate the Western blotting results, phospho-tyrosines on the membrane and the membrane itself were labeled and live-cell time frames were imaged by TIRF (Figure 5F). SHP2-KO BMDMs cultured on coated coverslips exhibited comparatively decreased fluorescence intensity of phospho-tyrosines, which revealed that SHP2 reshuffled phospho-tyrosine distribution during phagocytosis (Figure 5G).

**Figure 6:**
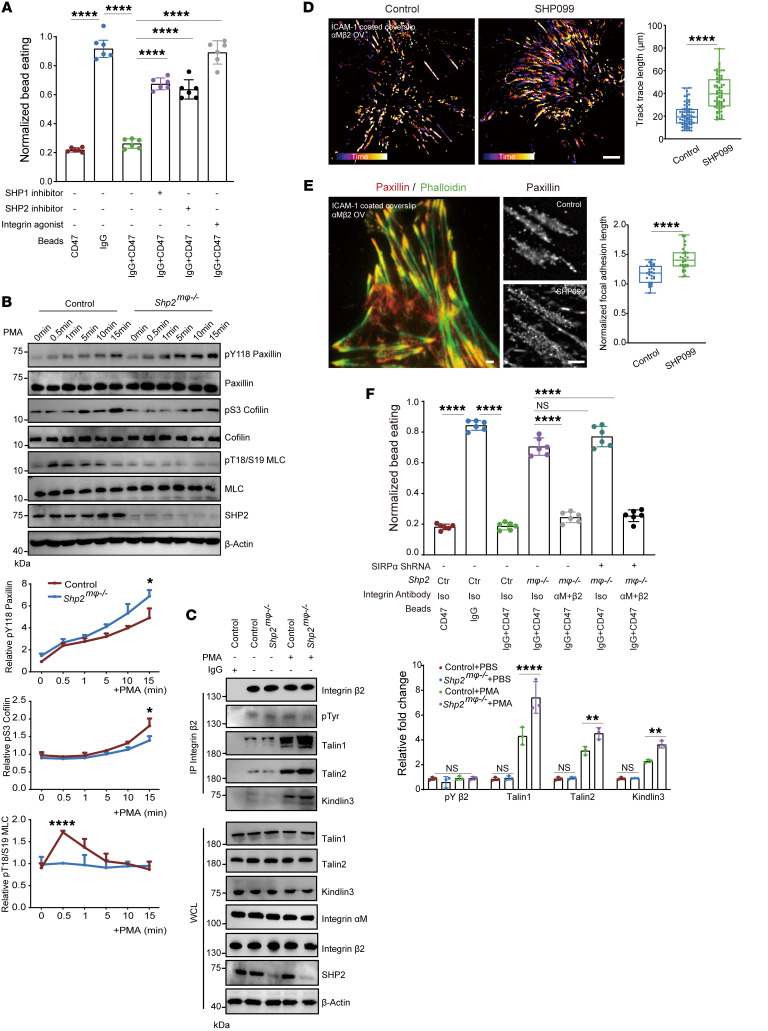
SHP2 deficiency activates α_M_β_2_ integrin to promote phagocytosis. (**A**) Normalized bead eating of indicated treated BMDM (*n* = 6). SHP1 inhibitor (TPI-1, 5 μM), SHP2 inhibitor (SHP099, 10 μM), integrin agonist (Mn^2+^, 1 mM). (**B**) Western blot indicating phosphorylated proteins in BMDMs treated with PMA (100 ng/ml) (*n* = 3). (**C**) Western blot indicating β_2_ integrin activation in BMDMs treated with PMA (100 ng/ml, 15 minutes) (*n* = 3). (**D**) Color-coded time-adherent trace of MEFs (cell number = 80). ICAM-1 (100 nM). Scale bar: 5 μm. (**E**) Left: representative TIRF images stained with F-actin (phalloidin) and paxillin of MEFs on ICAM-1–coated (100 nM) coverslip. Right: TIRF-STORM images of paxillin. Scale bars: 5 μm (cell number = 30). (**F**) Normalized bead eating of indicated BMDMs (*n* = 6). Anti-integrin antibody (10 mg/mL). Data are represented as mean ± SD. *P < 0.05; **P < 0.01; ****P < 0.0001; NS, *P* > 0.05. Two-tailed, unpaired Student’s *t* test (**B**, **D**, **E**); 1-way ANOVA followed by Tukey’s post hoc test (**A** and **F**); 2-way ANOVA followed by Tukey’s post hoc test (**C**).

According to the data mentioned above, we speculated that SHP2 was a target for inhibiting the CD47 checkpoint. Therefore, high-content screening microscopy was used to quantify the number of beads internalized by pretreated BMDMs (Supplemental Figure 5B). SHP1 inhibition, SHP2 inhibition, and integrin activation induced an increase in the engulfment of beads (Figure 6A). Recent work reported reactivation of α_M_β_2_ integrin bypassed CD47-mediated inhibition and rescued engulfment (20). Here, we hypothesized that SHP2 suppressed phagocytosis by primarily targeting integrin. Our data showed the aberrant phosphorylation state of the integrin-associated proteins paxillin, cofilin, and myosin light chains without α_M_β_2_ expression being disturbed in SHP2-deficient BMDMs upon PMA stimulation (Figure 6B and Supplemental Figure 5, C and D).

SHP2 has previously been shown to directly dephosphorylate FAK and vinculin (47), and colocalization images showed that SHP2 acts upon FAK and vinculin to regulate integrin-associated proteins (Supplemental Figure 5E). SHP2 deletion markedly enhanced the association between β_2_ integrin and Talin or Kindlin3, 2 critical components that sustain integrin activation by binding to the β_2_ tail (48) (Figure 6C). SHP2 deficiency increased the amount of GTP-Rap1 (49), which was positively correlated with integrin activity (Supplemental Figure 5F). Additionally, MEFs transfected with α_M_β_2_ were cultured on the coverslips coated with ICAM-1, which is the ligand for α_M_β_2_. TIRF-STORM showed a fast-maturing focal adhesion accompanied by a longer length in SHP099-pretreated MEFs (Figure 6, D and E). Importantly, the effects of functional blocking antibodies against α_M_ and β_2_ in suppressing engulfment were reversible with either SHP2 or SIRPα deficiency (Figure 6F). As deleting SHP2 had no effect on BMDMs engulfing antibody-opsonized beads (Supplemental Figure 5G), our data indicate that SHP2 in macrophages is a promising target for inhibiting the CD47 checkpoint and promoting ADCP.

### Neddylation inactivated SHP2 to promote macrophage-mediated engulfment of opsonized tumor cells.

**Figure 7:**
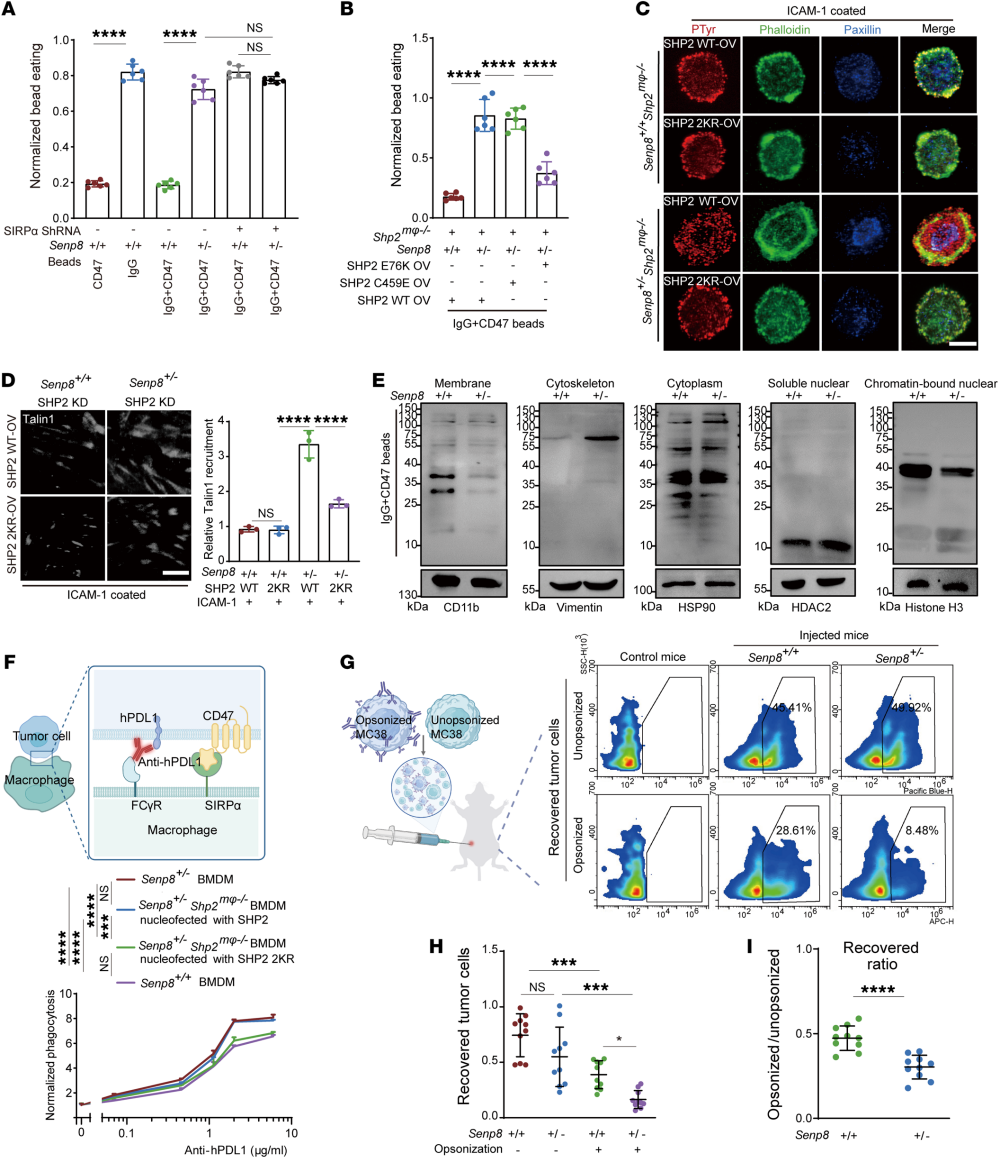
Neddylation of SHP2 promotes macrophage-mediated phagocytosis. (**A**) Normalized bead eating of BMDMs as indicated (*n* = 6). (**B**) Normalized bead eating of BMDMs as indicated (*n* = 6). (**C**) Representative TIRF images of BMDMs stained with pTyr, F-actin (phalloidin), and paxillin on ICAM-1–coated (100 nM) coverslips. Scale bar: 10 μm. (**D**) Representative TIRF images of MEFs stained with Talin1 on ICAM-1–coated (100 nM) coverslips (*n* = 6). Scale bars: 10 μm. (**E**) Western blot indicating tyrosine phosphorylation profile of subcellular fractionation of BMDMs. (**F**) Flow cytometry showed specific phagocytosis of hPDL1-expressing MC38 cells by indicated BMDMs (*n* = 3). (**G**–**I**) In vivo tumor cell recovery assay (*n* = 10). Schematic shows a 1:1 mixture of anti-mPDL1–opsonized or isotype–opsonized MC38 cells were injected i.p. into indicated mice. Peritoneal lavage fluid was required to calculate recovered tumor cells. Representative flow analysis plots of recovered tumor cells from mice (**G**). Data are represented as number of recovered tumor cells from mice (**H**). Data are represented as ratio between recovered tumor cells from mice that were differently opsonized (**I**). Data are represented as mean ± SD. *P < 0.05; ***P < 0.001; ****P < 0.0001; NS, *P* > 0.05. Two-tailed, unpaired Student’s *t* test (**I**); 1-way ANOVA followed by Tukey’s post hoc test (**A**, **B**, **D**, **F**, **H**).

To determine whether neddylation endows SHP2 with the ability to block SIRPα-mediated inhibitory signaling, *Senp8^+/–^* BMDMs were challenged with IgG- and CD47-coated beads. We concluded that SIRPα suppressed phagocytosis by specifically targeting neddylation, as SIRPα blockade relied on SENP8 to regulate macrophage engulfment (Figure 7A and Supplemental Figure 6A). In addition, constitutively activated mutants (SHP2 E76V) or catalytic-dead mutants (SHP2 C459E) were introduced into *Senp8^+/–^* *Shp2^mφ–/–^* BMDMs to further clarify the specific role of SHP2. It was found that SENP8 controlled macrophage engulfment in an SHP2 activity–dependent manner (Figure 7B). Our data also provided more evidence of neddylation in regulating SIRPα/SHP2 signaling. SHP2 2KR mutation restored paxillin and phospho-tyrosine localization in *Senp8^+/–^* *Shp2^mφ–/–^* BMDMs (Figure 7C). Promotion of Talin1 anchorage toward introduced integrin α_M_β_2_ was also abrogated by overexpressing neddylation-deficient SHP2 mutation in *Senp8^+/–^* SHP2 *K_D_* MEFs (Figure 7D). More importantly, *Senp8^+/–^* BMDMs presented a unique tyrosine phosphorylation profiling orchestrated by neddylation (Figure 7E). These data indicate SENP8 deletion relies on neddylated SHP2 to block the CD47/SIRPα axis.

Then, tumor-cell phagocytosis assay was conducted to verify our hypothesis. Due to the fact that macrophage-PD1 inhibits SHP2-mediated phagocytosis (50), we anticipated that the anti-PDL1 antibody would be more effective at enhancing ADCP in macrophages. Indeed, the treatment involving anti-PD1/PDL1 is also being used in clinical trials for certain CRC subtypes (51, 52). We replaced the antibody-opsonized beads with hPDL1-expressing MC38, which was opsonized at concentrations ranging from 0.1 to 10 μg/mL. Indeed, SENP8 deletion synergy with anti-hPDL1 treatment was found to rely on SHP2 K358 and K364 site neddylation (Figure 7F and Supplemental Figure 6B). The peritoneal tumor cell–killing model was utilized to assess the role of neddylated SHP2 in the CD47/SIRPα axis in vivo (Figure 7G). WT and SENP8 heterozygous mice were injected with a 1:1 mixture of anti-mPDL1–opsonized or isotype-opsonized MC38 cells. Subsequently, the remaining tumor cells of the isotype-opsonized group versus the antibody-opsonized group were quantified in peritoneal lavage fluids after 24 hours. Notably, SENP8 deletion achieved an enhanced tumoricidal effect in antibody-opsonized groups (Figure 7, H and I).

**Figure 8:**
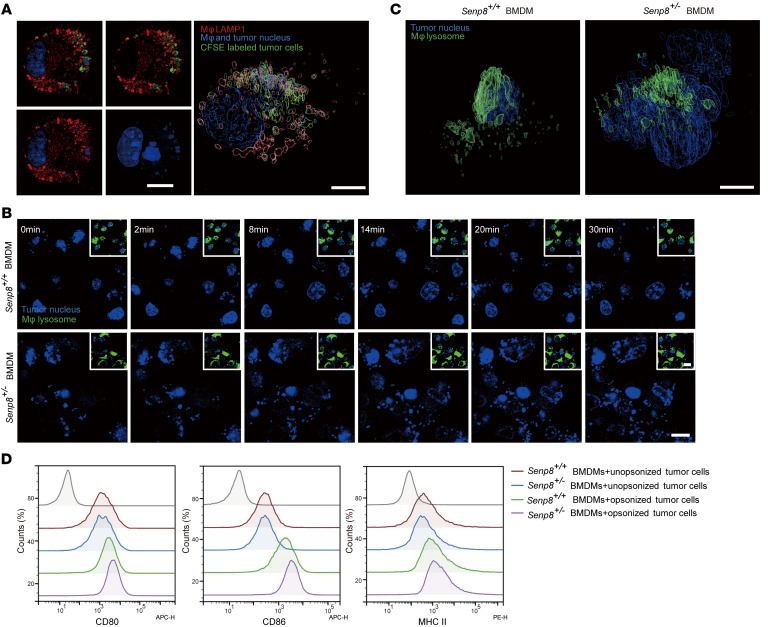
Neddylation of SHP2 in macrophages promotes clearance of tumor cells. (**A**) Confocal microscopy visualization of internalized tumor cells trapped in macrophage lysosomes and the corresponding reconstructed renderings by Imaris 9.5. Scale bars: 5 μm. (**B**–**C**) Tumor cell phagocytosis imaging. Representative time-lapse montage of labeled lysosomes of BMDMs treated with Hoechst 33342–labeled tumor cells as indicated. Scale bars: 10 μm (**B**). Corresponding reconstructed renderings by Imaris 9.5. Scale bars: 5 μm (**C**). (**D**) Representative flow analysis plots showed indicated surface expression of BMDMs. PDL1-opsonized MC38 cells were cocultured with BMDMs for 24-hour tumor cell digestion, after which BMDMs were detected by flow cytometry.

In addition to target cell recognition and cellular engulfment, macrophage-mediated engulfment of tumor cells also involves successive lysosomal digestion. Our data showed that internalized tumor cells were trapped in macrophage lysosomes, with their nuclei being fragmented and smeared (Figure 8A). Tumor cells cocultured with macrophages were also detected via live-cell imaging (Figure 8B). 3D rendering data demonstrated morphologically stronger nuclei transformation of opsonized tumor cells internalized by *Senp8^+/–^* BMDMs (Figure 8C). Furthermore, the digestion of MC38 cells triggered a general proinflammatory phenomenon of *Senp8^+/–^* BMDMs, which was revealed by increased CD86, CD80, and MHCII expression (Figure 8D).

### Neddylation of SHP2 synergizes with immunotherapy in vivo.

**Figure 9:**
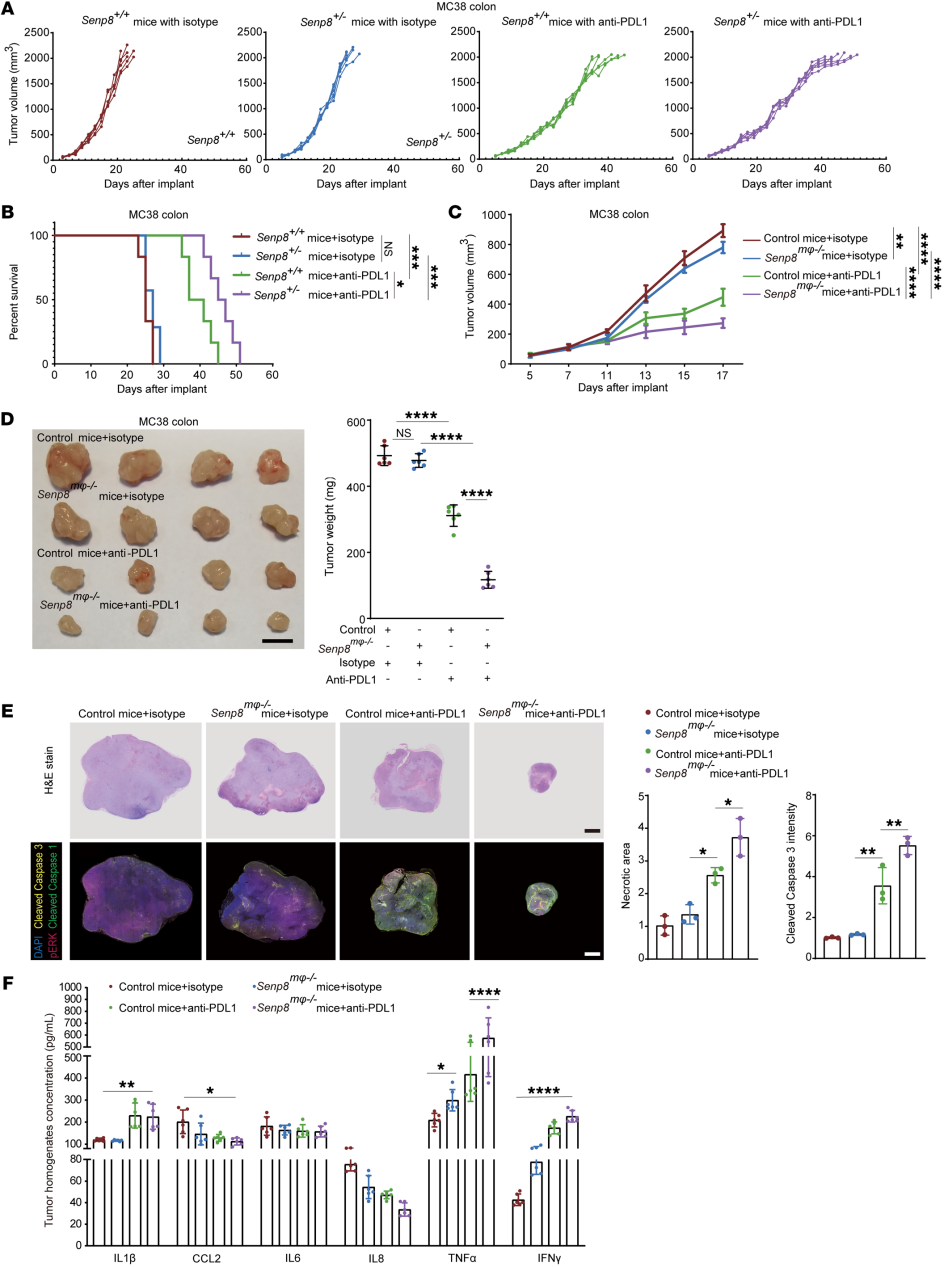
Neddylation induces SHP2 inactivation to potentiate the effect of immunotherapy in vivo. (**A** and **B**) Tumor sizes in the indicated mice (10 mg/kg anti-PDL1 antibody or isotype control, *n* = 6). Mice with tumor volumes of less than 2,000 mm^3^ are considered to be surviving (**A**). The survival of mice was monitored (**B**). (**C** and **D**) Tumor sizes of indicated mice (10 mg/kg anti-PDL1 antibody or isotype control, *n* = 6) (**C**). Tumor images and weights of indicated mice (10 mg/kg anti-PDL1 antibody or isotype control, *n* = 6). Scale bars: 10 mm (**D**). (**E**) Representative images of MC38 tumor sections (*n* = 3). H&E staining was conducted, and necrosis areas were measured. Fluorescent multiplex immunohistochemistry was conducted, and apoptosis was measured by cleaved caspase-3 staining. Scale bars: 1 mm. (**F**) ELISA analysis of MC38 tumor homogenates (*n* = 6). Data are represented as mean ± SD. *P < 0.05; **P < 0.01; ***P < 0.001; ****P < 0.0001; NS, *P* > 0.05. One-way ANOVA followed by Tukey’s post hoc test (**C**, **E**, **D**); 2-way ANOVA followed by Tukey’s post hoc test (**F**); Kaplan-Meier log-rank test (**B**).

To further investigate the therapeutic tumor control of targeting the CD47/SIRPα axis by SHP2 inactivation, syngeneic MC38 cells were implanted s.c. into the flanks of *Senp8^+/+^* and *Senp8^+/–^* mice. Of note, SENP8 deficiency enhanced the effect of PDL1 blockade on inhibition of tumor growth, which was accompanied by a marked increase in survival (Figure 9, A and B). To confirm the function of neddylated SHP2 in TIMs, macrophage-specific *Senp8^mφ–/–^* mice were generated (Supplemental Figure 6C). Tumors of *Senp8*^mφ–/–^ mice were identified as significantly sensitized to anti-PDL1 treatment (Figure 9C). Consistent with the delayed tumor growth, tumor weight was also significantly reduced in *Senp8^mφ–/–^* mice under PDL1 blockade (Figure 9D). H&E-stained sections of tumors from PDL1-blocking antibody–treated *Senp8^mφ–/–^* mice revealed markedly increased necrotic areas with scattered histiocytes (Figure 9E and Supplemental Figure 6D). Not surprisingly, multiplex IHC (mIHC) validated more profound pERK inhibition as well as increased cleaved caspase-3 signal in tumors from *Senp8^mφ–/–^* mice under anti-PDL1 treatment (Figure 9E and Supplemental Figure 6D), and SENP8 deficiency promoted M1 polarization of TIMs in tumor infiltrates (Supplemental Figure 6E). To provide additional support for SENP8 deficiency in a macrophage-reshaping tumor microenvironment, we also surveyed the composition of chemokines and cytokines. *Senp8^mφ–/–^* mice exhibited excess TNF-α and IFN-γ formation, which is a key factor of the inflammatory process in the tumor microenvironment (Figure 9F).

### The CD47/SIRPα axis requires SHP2 deneddylation to disrupt clearance of tumor cells.

**Figure 10:**
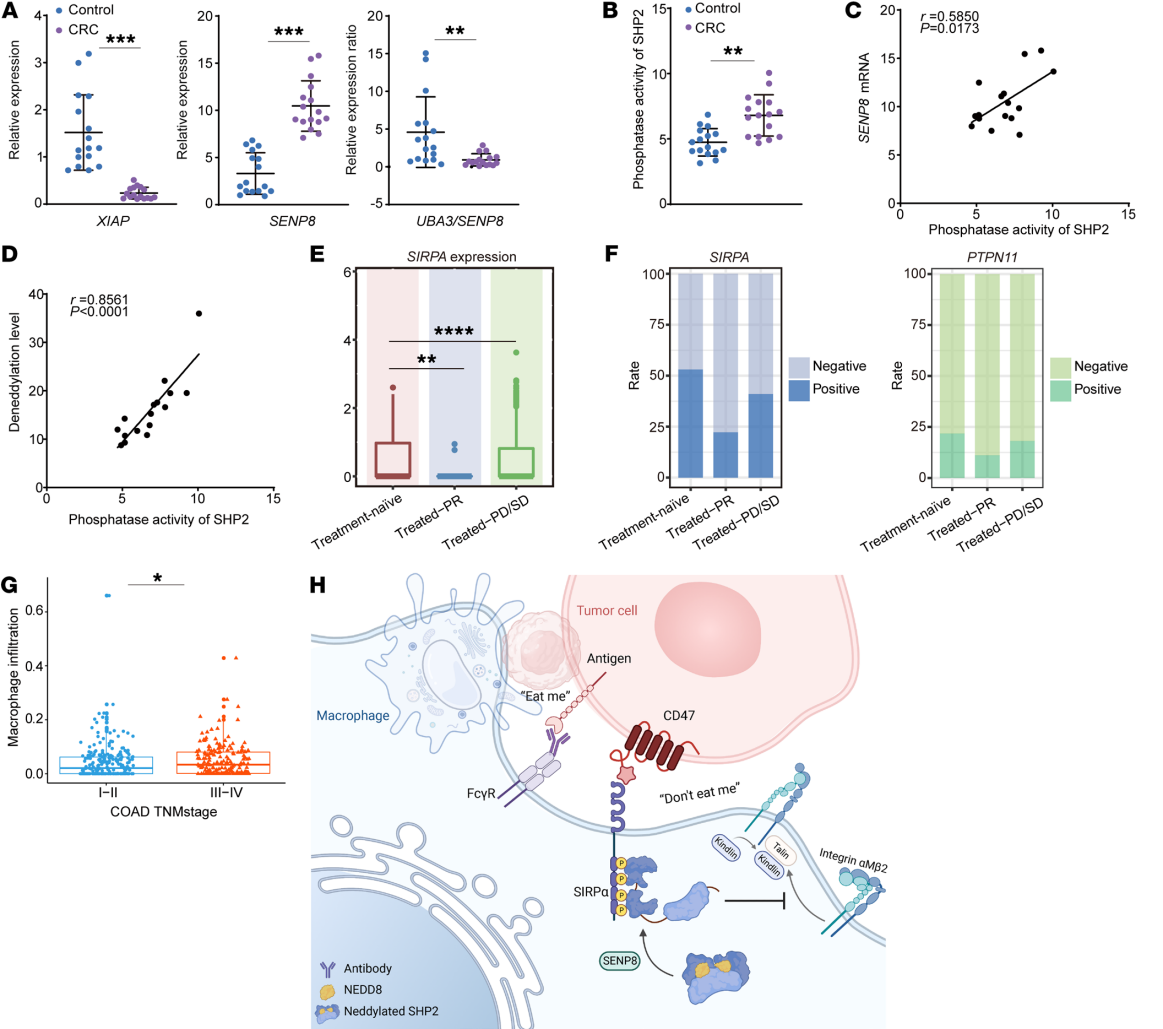
SHP2 activation in CD47/SIRPα axis of TIMs is related to the prognosis of CRC patients. (**A**–**D**) Macrophages of human tissue were collected (*n* = 16). mRNA levels are shown (**A**). SHP2 was IP with normalized protein concentration, and phosphatase activity was quantified (**B**). Correlations between *SENP8* mRNA and SHP2 phosphatase activity in TIMs (**C**). Correlations between deneddylation level and SHP2 phosphatase activity in TIMs (**D**). (**E**) Expression of SIRPα in tumor-infiltrating myeloid cells between CRC samples. *n* = 12 (NAC-treatment naive samples); *n* = 8 (NAC-treated PR samples); and *n* = 5 (NAC-treated PD/SD samples). (**F**) Rate of SIRPα^+^ and SHP2^+^ cells among TIMs. (**G**) Correlation of macrophage infiltration score and TNM stage of COAD cohort from the TCGA database. *n* = 234 (I and II); *n* = 183 (III and IV). (**H**) Proposed model of SHP2 deneddylation to ensure CD47/SIPRα signal. Data are represented as mean ± SD. *P < 0.05; **P < 0.01; ***P < 0.001; ****P < 0.0001. Two-tailed, unpaired Student’s *t* test (**A** and **B**); Pearson’s correlation (**C** and **D**); Wilcoxon’s test (**E** and **G**).

To demonstrate that SHP2 activation is associated with neddylation in vivo, we collected fresh tumor and paracancer tissue from microsatellite stable (MSS) CRC patients to compare neddylation states of SHP2 in suppressive TIMs and normal macrophages from intestinal mucosa. Varied mRNA levels of *SENP8* and *XIAP* in TIMs provided evidence of declining myeloid-SHP2 neddylation, and the *UBA3/SENP8* mRNA ratio of TIMs was largely outside the normal range (Figure 10A). The activity of SHP2 in TIMs was stronger than that in control cells of paired normal adjacent tissues, which suggests a reduction of SHP2 neddylation (Figure 10B). SHP2 activity in TIMs was positively correlated with deneddylation levels among MSS CRC patients, as measured by SENP8 mRNA or deneddylation enzyme activity (Figure 10, C and D).

Additionally, there appeared to be a correlation between the CD47/SIRPα axis in macrophages and the prognosis of CRC patients. Publicly available scRNA-Seq data of CRC patients treated with neoadjuvant chemotherapy (NAC) were analyzed (53) (Supplemental Figure 6F). The SIRPα expression of tumor-infiltrating myeloid cells exhibited a large decline in NAC-treated partial response (PR) groups and an increase in groups of NAC-treated patients with progressive disease (PD) or stable disease (SD) (Figure 10E). More specifically, in PR samples, there existed only a small fraction of SIRPα^+^ or SHP2^+^ macrophages, whereas an opposite trend was observed in PD/SD samples (Figure 10F). Further analysis reported a positive association between the score of macrophage infiltration and the tumor-node-metastasis (TNM) stage of the TCGA-COAD cohort (Figure 10G).

**Figure 11:**
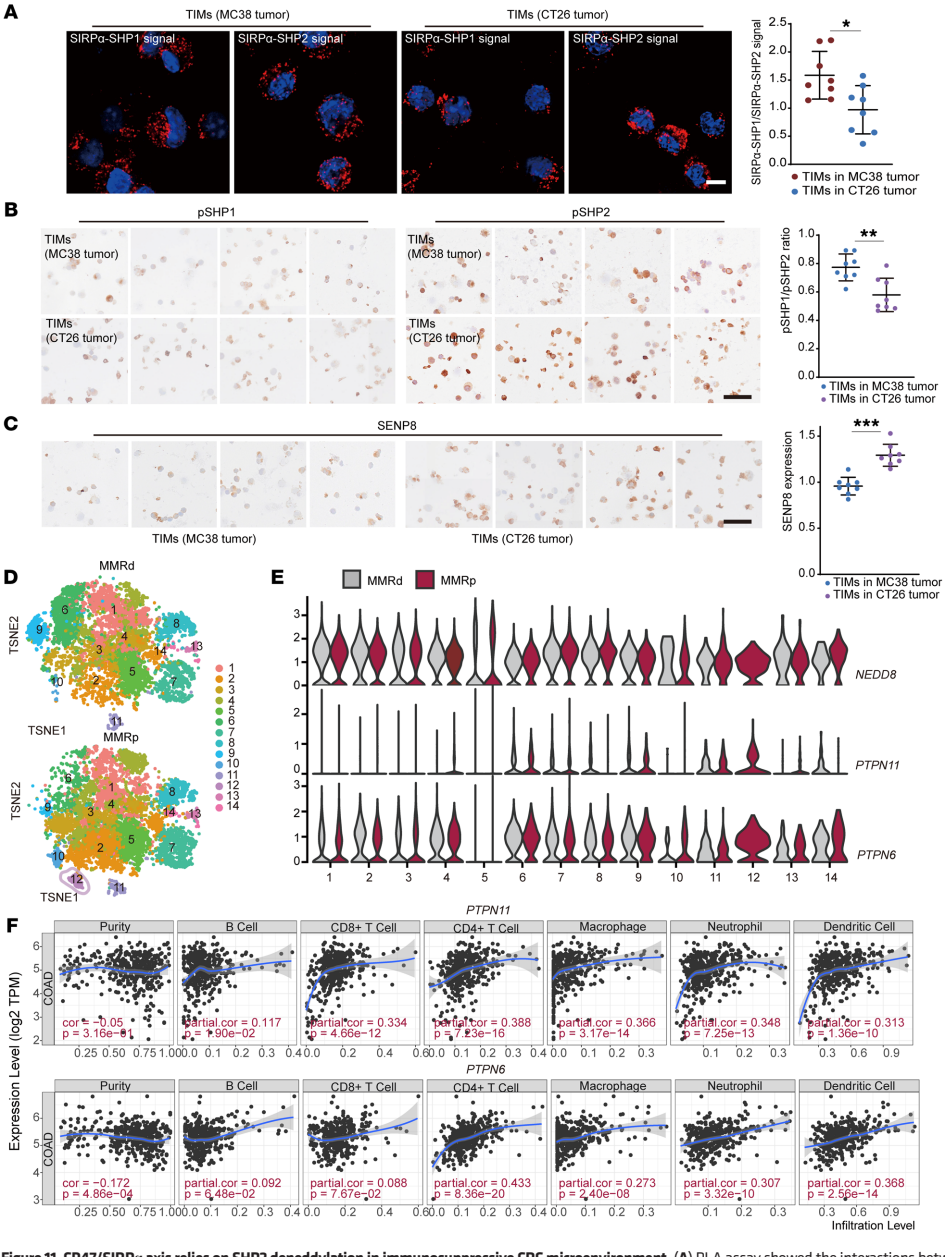
CD47/SIRPα axis relies on SHP2 deneddylation in immunosuppressive CRC microenvironment. (**A**) PLA assay showed the interactions between SIRPα and SHP1/SHP2 in indicated TIMs (*n* = 8). Scale bar: 10 μm. (**B**) pSHP1 (Y564) and pSHP2(Y542) expression in indicated TIMs (*n* = 8). Scale bar: 50 μm. (**C**) SENP8 expression in indicated TIMs (*n* = 8). Scale bar: 50 μm. (**D**) TSNEs of TIM clusters in MMRd and MMRp tumor samples. *n* =28 (MMRd samples); *n* = 34 (MMRp samples). (**E**) Violin plot shows expression levels of indicated genes of different clusters in MMRd and MMRp tumor samples. *PTPN11* encodes SHP2; *PTPN6* encodes SHP1. (**F**) Association analysis of *PTPN11* or *PTPN6* gene expression and immune cell infiltration in COAD cohort from the TCGA database. Data are represented as mean ± SD. *P < 0.05; **P < 0.01; ***P < 0.001. Two-tailed, unpaired Student’s *t* test (**A**–**C**); partial correlation (**F**).

As SHP1 and SHP2 demonstrate a broad specificity downstream of SIRPα, it is urgent to explain the specific roles of SHP2 in the TIMs of CRC. Clinical outcomes with PD1/PDL1 inhibition were encouraging in CRC patients whose tumors were mismatch repair–deficient (MMRd)/microsatellite instability-high (MSI-H) (54), while responses were rarely seen with mismatch repair–proficient (MMRp)/MSS tumors (55, 56). We isolated TIMs from MC38 and CT26 s.c. tumors, which were characterized as MSI-H and MSS CRC, respectively. Then, proximity ligation assay (PLA) was conducted to detect the interactions between SIRPα and these 2 phosphatases, and the signal of interaction was visualized and quantified as discrete spots. SHP2-mediated signaling exhibited relative prevalence in macrophages of CT26 tumors (Figure 11A). Furthermore, pSHP1 (Y564) and pSHP2 (Y542) expression were compared using immunocytochemistry, as the phosphorylation state is thought to be a form of activation for both proteins. The calculated pSHP1/pSHP2 ratio was dramatically lower in CT26 tumors than in MC38 tumors (Figure 11B). And the immunocytochemistry result showed increased SENP8 expression in TIMs of CT26 tumors compared with MC38 tumors (Figure 11C). Subsequently, we also analyzed macrophages in untreated CRC patients (57) (Supplemental Figure 7A). The predominant change in the macrophage composition of MMRd versus MMRp tumors was in cluster 12, which was identified using the term “cell junction disassembly” in GO analysis (Figure 11D and Supplemental Figure 7B). Cells in cluster 12 in MMRp tumors were identified as CX3CR1^+^ macrophages, which possess the highest transcription levels of immune-inhibitory receptor genes, including *SIRPA*, *CFS1R*, and *MERTK* (Supplemental Figure 7C). MerTK has been noted in the removal of dying cells, and blockade of MerTK resulted in elevated tumor control (58). Gene expression analysis identified the distinct macrophage population of cluster 12 with low expression of *NEDD8* (Figure 11E). Notably, cluster 12 showed the highest gene transcript levels for *PTPN11* over the other clusters, but not for *PTPN6* (Figure 11E). Then, TIMER2.0 (http://timer.cistrome.org) was used to analyze the correlation of *PTPN6/PTPN11* mRNA levels with the infiltration of immune cells based on the TCGA-COAD cohort. The results indicated that *PTPN11* expression had a higher positive correlation with immune cell infiltration, especially in macrophages (Figure 11F). These results emphasize the SHP2-mediated immunosuppressive signal in CRC.

### Combination therapy with the allosteric SHP2 inhibitor and anti-PDL1 treatment overcomes the immunosuppressive microenvironment of CRC.

**Figure 12:**
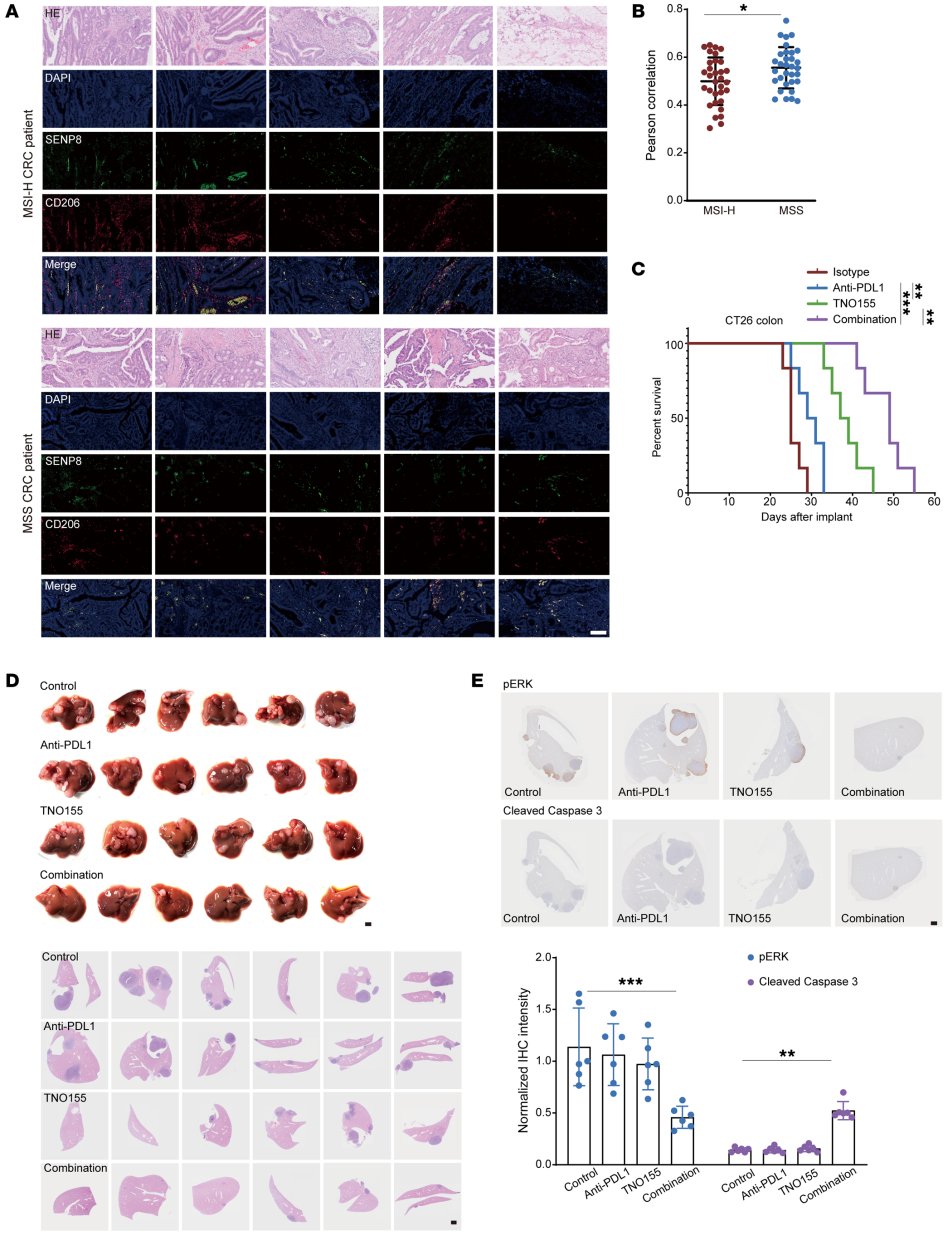
SHP2 inhibition synergizes with immunotherapy to disrupt the immunosuppressive CRC microenvironment. (**A**) Immunofluorescence images of MSI-H CRC and MSS CRC (*n* = 32) staining indicated markers. Scale bars: 100 μm. (**B**) Pearson’s correlation of SENP8 and CD206 shown in Figure 8A. (**C**) TNO155 (15 mg/kg, daily intragastric administration from day 5), anti-PDL1 (10 mg/kg, i.p. from day 5 every other day), or a combination of both in CT26 s.c. tumor-bearing mice. Mice with tumor volumes less than 2,000 mm^3^ are considered as surviving, and survival of mice was monitored (*n* = 6). (**D**) Spleen images and H&E staining from the spleen-liver metastasis model of indicated groups (*n* = 6). Scale bars: 1 mm. (**E**) IHC staining from the spleen-liver metastasis model of indicated groups (*n* = 6). Data are represented as mean ± SD. *P < 0.05; **P < 0.01; ***P < 0.001. Two-tailed, unpaired Student’s *t* test (**B**); Kaplan-Meier log-rank test (**C**); 2-way ANOVA followed by Tukey’s post hoc test (**E**).

Immunosuppressive TIMs in human colon cancer tissue were further identified as related to SENP8 using stained paraffin slides. The immunofluorescence images of MSI-H CRC and MSS CRC tumors revealed higher SENP8 expression in CD206^+^ cells. Importantly, a higher correlation existed between SENP8 and CD206 in TIMs of MSS CRC patients (Figure 12, A and B). Given that SHP2 allosteric inhibitor TNO155, which is developed from SHP099, has been employed in clinical trials of KRAS^mutant^ solid tumors (59), we investigated to determine whether SHP2 inhibition led to a better treatment outcome in MSS CRC. Indeed, *PTPN11* mRNA expression was also upregulated in COAD samples from the TCGA-COAD cohort (Supplemental Figure 7D). CT26 cells with the KRAS^G12D^ mutation belong to the MSS-type mouse colorectal tumor cell line and are nonsensitive to immune checkpoint blocking. We then examined the antitumor efficacy of TNO155 and the anti-PDL1 antibody and their combination in CT26 s.c. tumor models. In comparison with anti-PDL1 antibodies, TNO155 inhibited CT26 tumor growth more effectively. The combination of TNO155 and the anti-PDL1 antibody demonstrated robust antitumor benefit, as evidenced by a marked increase in the time to reach endpoint tumor burden (Figure 12C). As CRC frequently metastasizes, we also implanted CT26 cells into the spleen to establish a spleen-liver metastasis model. Then, mice were treated with TNO155 and anti-PDL1 antibody and a combination of the two from 1 month after implantation. H&E staining showed that TNO155 combined with the anti-PDL1 antibody significantly reduced liver metastasis compared with other treatments (Figure 12D). The significant antitumor benefit of combination treatment was evidenced by pERK1/2 and cleaved caspase-3 expression (Figure 12E).

Taken together, our data demonstrate that the CD47/SIRPα axis requires deneddylation to activate SHP2 (Figure 10H). The inhibition of macrophage-specific SHP2 disrupts the immunosuppressive microenvironment of CRCs and improves their response to immunotherapy. In particular, treatment supplementation with SHP2 inhibitors overcomes immunotherapy resistance in MSS CRC. Our study highlights the importance of combining SHP2 targeting with other CRC therapies.

## Discussion

The number of ongoing clinical trials of CD47 and SIRPα antagonists is increasing as strong emerging evidence showed CD47/SIRPα axis blockage promotes tumor control. Here, we identify SHP2 as a checkpoint of the CD47/SIRPα axis. More specifically, its modified form, neddylated SHP2, is required for reinvigorating immunosurveillance of macrophages to eradicate tumors.

Our data characterized SHP2 as a genuine non-Cullin substrate that met the proposed criteria (24). Amino acid sequence alignment of SH2 domain–containing phosphatases specifically highlighted K358 and K364 of SHP2. Indeed, the K364 residue that is reported as positively charged patches has been shown to be critical for SHP2 conformational transition (60). Importantly, crystallographic data and MD data of 2 different SHP2 inhibitors identified K364 as an interacting residue that is in contact with the inhibitor compound (61, 62). As neddylation-impaired SHP2 binding to phospho-ligand also requires K364 to be modified, we believe our findings provide new clues to the structural understanding of the SH2-PTP interface. SUMOylation (63), phosphorylation (64), and oxidation (65) have been shown to contribute to SHP2 enzyme activity, but to our knowledge, SHP2 activation tuned by neddylation has never been reported. Accordingly, the latest work established a “multiple gear” regulatory mechanism of SHP2 (66), in which different activators, oncogenic mutations, and allosteric inhibitors shift SHP2 conformational equilibrium among the inactive, semi-active, and full-active states, regulating SHP2 activity to different levels. As SHP2 coding variants manifested aberrant neddylation, we concluded that neddylation played a nonredundant role in regulating SHP2 activity.

We also demonstrated a sensitive neddylation profile of tumor-infiltrating monocytes/macrophages downstream of SIRPα, which validated that neddylation participated in the reshaping of the tumor immune microenvironment. Neddylation E1 inhibitor MLN4924 has been used in clinical trials to inactivate Cullin-RING E3 ligase (CRL), thereby interrupting protein homeostasis and subsequently leading to cancer cell death (67). However, MLN4924 failed to boost event-free survival (EFS) of patients in a phase III leukemia trial (ClinicalTrials.gov NCT03268954). SHP2 is frequently mutated in acute myeloid leukemia (AML) as well as in juvenile myelomonocytic leukemia (JMML), and SHP2 inhibition was established as reducing leukemogenesis in models of genetic or epigenetic mutations (68, 69). In addition, catalytic cysteines of SENP8 have been reported to be sensitive to ROS, which dynamically influence the tumor microenvironment (70). As MLN4924 simultaneously inactivates CRLs and activates SHP2, this paradox validates further coordination between inhibiting total neddylation and targeting the predominant substrate.

Recently, PD1/PDL1 blockade treatment has proven to be an effective immunotherapeutic strategy for MSI-H CRC patients. It is reasonable for MSI-H CRC patients to benefit from the immunotherapeutic strategy, as higher tumor mutation burden and neoantigen load are crucial for the immune activation to the potentiate antitumor activity (71). However, CRC is highly heterogeneous, and approximately 75% of CRC patients display high-level MSS; thus, the primary goal is to establish antitumor immune response in the context of limited immunogenicity (72). Although SIRPα is conservatively expressed on myeloid cells rather than lymphoid cells, our CyTOF data for tumor-infiltrating immune cells exhibited a detectable PD1 loss in T cell subsets (Supplemental Figure 1F) as well as increasing amounts of B cells (Figure 1D) after CD47/SIRPα axis blockage, which demonstrates TIMs as restrictive factors in bridging of innate and adaptive immunity. Furthermore, our data showed that MSS induces expansion of an immune-suppressive distinctive SIRPα^hi^SHP2^hi^NEDD8^lo^ macrophage population. We also observed that dual inhibiting of PDL1 and SHP2 achieved better tumor control of MSS CRC. Our findings here show the promising value of blocking the CD47/SIRPα axis to replenish immune mobilization in the tumor microenvironment, which is of clinical benefit for improving the low treatment response rate of CRC.

Besides the PD1/PDL1 (73) axis and the CD47/SIRPα axis, recently reported CD24 (74) signaling through macrophage Siglec-10 further emphasizes an SHP2-regulated hub downstream of immune receptors. As clinical trials of SHP2 allosteric inhibitors emerge, our finding of SHP2-mediated immunosuppression in colorectal tumorigenesis lays the foundation for combining immune checkpoint inhibitors and the SHP2 inhibitor in CRC immunotherapy.

## Methods

Full methods are available in Supplemental Methods.

### Animals.

C57BL/6 *Senp8^fl/fl^* mice were obtained from GemPharmatech Co. C57BL/6 and C57BL/6 *Senp8^+/–^* mice were obtained from Beijing Viewsolid Biotech Co. *Shp2^fl/fl^* mice were a gift from Gen-sheng Feng (UCSD, La Jolla, California, USA), and *Ube2m^fl/fl^* as well as *Ube2f^fl/fl^* mice were gifts from Sun Yi (Zhejiang University). Lysm Cre/+ mice were mated respectively with *Shp2^fl/fl^*, *Ube2m^fl/fl^*, *Ube2f^fl/fl^*, and *Senp8^fl/fl^* mice. *Senp8^+/+^* and *Senp8^+/–^* mice were crossed with Lysm Cre/+ *Shp2^fl/fl^* mice to obtain SHP2-deleted and SENP8-disrupted BMDMs. Littermates were randomized to different groups in which each cohort of animals received different treatments. Mice were kept under specific pathogen–free conditions. Mouse identification primer sequences are listed in the Supplemental Table 1.

### Cells.

HEK293T and THP1 cells were purchased from the Chinese Academy of Sciences. MC38 and CT26 cells were purchased from ATCC. HEK293T, MC38, and CT26 cells were grown in DMEM basic medium (Gibco, Thermo Fisher Scientific) supplemented with 10% FBS (Gibco, Thermo Fisher Scientific). Thp1 cells were grown in RPMI 1640 medium (Gibco, Thermo Fisher Scientific) supplemented with 10% heat-inactivated FBS (Gibco, Thermo Fisher Scientific). To generate BMDMs, bone marrow was flushed from tibias and femurs; then, bone marrow cells were plated in RPMI 1640 medium (Gibco, Thermo Fisher Scientific) supplemented with 10% heat-inactivated FBS (Gibco, Thermo Fisher Scientific) and GM-CSF (10 ng/ml) for 6 days. To generate mouse embryonic fibroblasts (MEFs), pregnant female mice were sacrificed and the embryo detached from the placenta. After removal of the head, tail, limbs, and internal organs, the remains were digested to obtain single isolated cells. MEF were cultured in DMEM basic medium (Gibco, Thermo Fisher Scientific) supplemented with 10% FBS (Gibco, Thermo Fisher Scientific). All cells were maintained in a humidified atmosphere containing 5% CO_2_ at 37°C.

### Transfection, lentivirus knockdown, and CRISPR/Cas9 knockout.

HEK293T, MC38, and MEF cells were transfected with plasmids using LIPO3000 (catalog L3000015; Invitrogen, Thermo Fisher Scientific) according to the manufacturer’s instructions. BMDMs were transfected with plasmids or siRNA using the Mouse Macrophage Nucleofector Kit (Lonza) according to the manufacturer’s instructions. HEK293T cells were transfected with siRNA using INTERFERin (Polyplus) according to the manufacturer’s instructions. Mouse SHP2 or SIRPα knockdown (SHP2 *K_D_* MEFs and SIRPα *K_D_* BMDMs) was performed by lentiviral expression shRNA. The gRNA was used to generate SENP8-KO HEK293T cells. gRNA sequences were cloned into the LentiCRISPR, version 2, according to a standard protocol. gRNA/Cas9 expression plasmids were transfected, and then cells were selected with puromycin (2 μg/ml); the resulting single colonies were picked and expanded. Related sequences are listed in Supplemental Table 1.

### Mouse tumor models and treatment.

Mice were s.c. injected with 1 × 10^6^ MC38 cells. Tumors were allowed to form for 5 days, and then mice were injected i.p. with 10 mg/kg anti-PDL1 antibody (Bio X Cell) or isotype control at the peritumoral site every other day; tumor size was measured using calipers. Treatment was continued for 12 days, after which mice were euthanized and tumors were harvested for further analysis. Tumor volume was calculated according to the following formula: length × width^2^ × 0.5. For monitored survival, mice with tumor volumes of less than 2,000 mm^3^ were considered to be surviving. For conducting CyTOF, tumors were allowed to form for 5 days, and mice were then injected s.c. with 2 mg/kg Pep-20 or normal PBS as the negative control at the peritumoral site every day. Treatment was continued for 2 weeks.

For CT26 s.c. tumors, experimental operations were the same as in the steps mentioned above. Daily intragastric administration of 15 mg/kg TNO155 (Selleck Chemicals) was performed by gavage needle from day 5, and 10 mg/kg anti-PDL1 antibodies were injected i.p. at the peritumoral site every other day from day 5. Six-week-old mice were used for spleen-liver metastasis assay; 4 × 10^5^ CT26 cells were injected into spleens when mice were under anesthetic. After 1 month, mice were treated as described in the CT26 s.c. tumor model for 10 days. Mice were then euthanized, and livers were separated for histological analysis.

### Western blotting, IP, and co-IP.

For Western blotting assay, cells were lysed in SDS buffer on ice and boiled for 10 minutes at 100°C. Samples were resolved with SDS-PAGE, which was followed by transfer onto nitrocellulose membranes (Pall). Samples were probed with the indicated primary antibodies as well as secondary antibodies.

For IP of the neddylated proteins, cells were lysed in 1% SDS at 100°C for 5 minutes, then diluted with IP lysis buffer (50 mM Tris-HCl, 150 mM NaCl, 0.5%NP-40, 40 mM sodium pyrophosphate,1 mM Na_3_VO_4_, and protease inhibitors, pH 7.5) to reach 0.1% of the final SDS. The lysate was incubated with antibody-conjugated magnetic beads overnight at 4°C. After that, beads were washed and eluted with 1× SDS loading buffer for Western blot analysis. For co-IP assays, cells were lysed in co-IP lysis buffer (50 mM Tris-HCl, 5 mM EDTA, 150 mM NaCl, 0.5% [v/v] NP40, 10% glycerol, 1 mM PMSF, 1 mM Na_3_VO_4_, and 10 mM NaF, pH7.5). Lysate was incubated with antibody-conjugated magnetic beads overnight at 4°C. After that, beads were washed and eluted with 1× SDS loading buffer for Western blot analysis. Specific secondary antibodies were used to avoid light/heavy-chain signal interference. Relative antibodies are listed in Supplemental Table 1.

### Human samples.

Tumor biopsy samples of 16 patients and paraffin sections of 64 patients were obtained. The baseline characteristics of all participants are shown in Supplemental Table 2. Tumor biopsy specimens were collected from 7 patients, and diagnosis was based on clinical, endoscopic, and histological criteria. The baseline characteristics of all participants are shown in Supplemental Table 3.

### Statistics.

Statistical analysis was performed using Prism software (GraphPad, version 8.0). All quantitative data are presented as mean ± SD. Unpaired Student’s *t* tests were used for comparisons between 2 groups. Multiple-group comparisons were conducted by 1-way or 2-way ANOVA followed by Tukey’s post hoc tests. *P* < 0.05 was considered statistically significant.

### Study approval.

All animal experiments were approved by the Animal Care and Use Committee of the Zhejiang University School of Medicine (ZJU20220104). The protocols for the collection of human tissue in the study were approved by the Ethical Committee of Sir Run-Run Shaw Hospital (no. 20180226-51). The use of biopsy material was approved by the Ethics Committee of The First Affiliated Hospital (no. 2017-257). This study was performed in compliance with all relevant ethical regulations, and all participants provided informed consent.

## Author contributions

YK, XZ, and YL conceived the study and designed the experiments. YL and HZ performed most of the experiments, assisted by PL and Y Shi. DL, BT, WX, J Zhou, JX, TZ, ZJ, J Zhang, KY, YZ, Y Sun, and HC provided funding, technical, and material support. YL performed bioinformatics analysis. YL and HZ analyzed the data and wrote the manuscript. All authors reviewed the manuscript and provided final approval for submission.

## Supplementary Material

Supplemental data

## Version 1 01/10/2023

In-Press Preview

## Version 2 02/15/2023

Electronic publication
