# Supplementary material for: SHP2 deneddylation mediates tumor immunosuppression in colon cancer via the CD47/SIRPα axis
Source: J Clin Invest. 2023 Feb 15;133(4):e162870. doi: 10.1172/JCI162870 (PMC9927946; doi:10.1172/JCI162870)
Supplement: Supplemental data [file jci-133-162870-s194.pdf]

Supplemental Materials for

SHP2 deneddylation mediates tumor immunosuppression in colon cancer *via* the CD47/SIRPα axis

Yiqing Li[1], Hui Zhou[1], Pan Liu[1], Dandan Lv[2], Yichun Shi[1], Bufu Tang[3], Jiaqi Xu[4], Tingting Zhong[4], Wangting Xu[5], Jie Zhang[6], Jianying Zhou[5], Kejing Ying[2], Yongchao Zhao[7], Yi Sun[7], Zhinong Jiang[4], Hongqiang Cheng[8], Xue Zhang[1*], Yuehai Ke[1*]

*Correspondence to: yke@zju.edu.cn or zhangxue@zju.edu.cn.

This PDF file includes:

Supplemental Methods

Supplemental Figures 1 to 7

Supplemental Tables 1 to 4

Supplemental Methods

Reagents

The information on all the reagents used in this article was listed in the Supplemental Table 1.

Expression vectors

To construct SIRPα, SHP2, NSH2, CSH2, PTP, 2SH2, SHP1, NEDD8, SENP8, UBE2M, UBE2F, Cbl-c, c-Cbl, MDM2, TRIM4, XIAP, RBX1, CD11b, CD18, LAMP1, PDL1, the corresponding human full-length sequences were cloned and inserted into the PXJ40 expression vectors.

NEDD8 K11R, NEDD8 K48R, SHP2 K358R, SHP2 K364R, SHP2 K358R/ K364R, SHP2 D61G, SHP2 A72G, SHP2 E76K, SHP2 E139D, SHP2 N308D, SIRPα 4YF(Y429F/Y452F/Y470F/Y476F), and SHP2 R32A/R138A PXJ40 vectors were produced by base mutation in cDNA encoding sequences.

To produce SRC, XIAP, SHP2 WT, SHP2 D61G, SHP2 E76K, SHP2 K358R/ K364R, and SHP2 K364R recombinant protein, the corresponding human sequences were cloned and inserted into the Pcold-GST plasmids. All constructs were verified by sequencing.

Analysis of public scRNA-seq datasets

ScRNA-seq data of tumor-infiltrating myeloid cells from different human tumors were acquired accompanying cluster annotations, they were downloaded from the Gene Expression Omnibus (GEO) with accession codes GSE46771 and GSE154763. Harmony algorithm is used for performing integration of single-cell genomics datasets,

and variable genes across monocyte and macrophage clusters were computed. ScRNA-seq data from tumor single-cell suspensions between MMRd and MMRp subtypes in colorectal cancer patients were downloaded from GEO with accession code GSE178241. Firstly, the processed data from the database was extracted. Seurat 3.1 was used for quality control and expression level normalization and normalization and clustering of cells. Macrophage populations were extracted for further analysis. After TSNE dimensionality reduction, GO was used for gene differential enrichment analysis. The differential genes were logfc.threshold = 0.25, and the top 100 genes with differential values were used for GO analysis. GO entries take the top 10 visualizations. ScRNA-seq data from tumor-infiltrating myeloid cells from patients with MSS colorectal cancer receiving NAC were downloaded from the author's website http://www.cancerdiversity.asia/scCRLM. The data are reproduced according to the standards of the article.

Flow sorting of TIMs

The tumor was minced and digested in DMEM containing 300 units /mL collagenase IV (Worthington) and 50 U/ml DNase I (Sigma) at 37°C for 1 h. Red blood cells are removed by ACK Lysing Buffer. The cell mixtures were then filtered through 75 mm cell strainers. All the TIMs were isolated by flow sorting. The panel contains CD45-APC, CD68-FITC, and live/dead stains.

Organoids

Patient-derived tumor organoids were cultured by air-liquid interface methods. Human tissues from resected tumors were minced finely on ice, washed twice in ADMEM/F12

(Gibco), resuspended in 1mL of a mixture of Type I collagen gel (Trevigen) containing 10X Ham's F12 (Thermo) and reconstitution buffer (2.2 g NaHCO3 in 100 ml, 0.05 N NaOH, 200 mM HEPES) in a ratio of 8:1:1, respectively. Next, the fragment-collagen solution was added on top of a 0.4 μm transwell insert (PICM03050, Millicell-CM, Millipore), which was previously coated with 1 ml of the mentioned solution, The transwell containing tumor tissue and collagen was placed into an outer 60 mm cell culture dish containing 1.0 mL of ADMEM/F12 supplemented with 50% Wnt3a, R-spondin1 conditioned medium as well as HEPES (Gibco), Glutamax (Gibco), Nicotinamide (Sigma), N-Acetylcysteine (Sigma), B-27 without vitamin A (Gibco), A83-01 (Tocris), Penicillin-Streptomycin (Gibco), Gastrin (Tocris), SB-202190 (Sigma), hIL-2 (Novoprotein) and EGF (Novoprotein). Organoids were passaged by dissociation with 300 units /ml collagenase IV (Worthington) at 37°C for 30 min and replated at the desired density.

***In vitro* neddylation assay**

Human XIAP, SHP2 WT, SHP2 D61G, SHP2 Y62D, SHP2 E76K, SHP2 K358R/ K364R, and SHP2 K364R were purified in-house. Relative recombinant proteins were incubated with components of the NEDD8 Conjugation Initiation Kit (Boston Biochem) for 1h at 37°C. Samples were separated on SDS-PAGE and analyzed by immunoblotting.

Recombinant protein purification

GST-tagged fusion proteins were purified from BL21 cells. Cells were grown in constant shaking (220 rpm) at 37°C until the log phase, then induced with 0.1 mM IPTG

at 16°C for 12 h. Cells were harvested, resuspended in lysis buffer (20 mM Tris-HCL PH 7.5, 300 mM NaCl, 1% Triton X-100, and Protease Inhibitor Cocktail), and sonicated. Cell debris was removed by centrifugation and supernatant was incubated with glutathione-sepharose overnight at 4°C. The bound proteins were cleaved with HRV 3C protease for the fusion tag.

SHP2 full length and PTP domain enzyme activity

The catalytic activity of neddylated SHP2 or PTP was monitored using the surrogate substrate DiFMUP. Overexpressed Myc-SHP2 or Myc-PTP Cells were lysed in Co-IP lysis buffer without phosphatase inhibitor and incubated with Myc antibody-conjugated magnetic beads. Beads were finally kept in assay buffer (60mM HEPES, pH 7.2, 75mM NaCl, 75mM KCl, 1mM EDTA, 0.05% P-20, 5mM DTT). SHP2 bonded beads need extra incubation with 1μM 2P-IRS-1 (H2N-LN (pY) IDLDLV (dPEG8) LST (pY) ASINFQK-amide) for 30 min incubation at 25 °C. Then, substrate DiFMUP was added and reacted at 25 °C for 30min. The reaction was then quenched, and the fluorescence signal was monitored using excitation and emission wavelengths of 340 nm and 450 nm on a microplate reader (M5, Molecular Devices).

Immunofluorescence staining and confocal microscopy

For immunofluorescence staining, cells were fixed in 4% PFA (pH7.0) and permeabilized with 0.5% Triton X-100 before blocking with 4% goat serum. Samples were incubated with primary antibody followed by secondary antibody incubation and then mounted in an antifade mounting medium with DAPI. The samples were imaged using an Olympus IX83-FV3000 confocal microscope (Olympus).

Total internal reflection fluorescence microscopy

TIRF microscopy was conducted on a Nikon N-STORM & A1 Cell TIRF system with a DU897 EMCCD 100Xoil TIRF objective and the fluorescence image were collected with Nis-Elements software (Nikon). BMDMs and MEFs were removed from their culture dish using 5% EDTA in PBS, washed, and resuspended in the HEPES imaging buffer (20 mM HEPES, 135 mM NaCl, 4 mM KCl, 10 mM glucose, 0.1 mM $Cacl_2$, 0.1 mM $Mgcl_2$) before being added to the coated TIRF chamber. After 30 min interaction with specific coating ligands, BMDMs were stained for analysis, MEFs need 1h interacting. For live-cell imaging, BMDMs were labeled with Wheat Germ Agglutinin (Thermo) and Alexa Fluor-488 Phospho-Tyrosine antibody (CST) before being added to the coated TIRF chamber. Cells were imaged on a heated stage and supplemented with warmed (37°C) humidified air. Timeseries images were analyzed with ImageJ software.

Stochastic optical reconstruction microscopy (STORM) image acquisition

STORM microscopy was conducted on a Nikon N-STORM & A1 Cell TIRF system with a DU897 EMCCD 100Xoil TIRF objective and the fluorescence image was collected with a Nikon N-STORM super-resolution system (Nikon Instruments Inc.) Besides required channels, a 405 nm laser was used to increase the number of on-state fluorophores according to generally recommended. Cells were stained and then immersed in STORM imaging buffer, and Nikon microscopic imaging device provided a Perfect Focus System (PFS) to achieve real-time correction of focus drift in the Z-axis direction. For live MEF imaging, MEFs were treated with SHP099 for 4h and then

labeled with Wheat Germ Agglutinin (Thermo) before being added to the ICAM-1 coated TIRF chamber. Cells were imaged on a heated stage and supplemented with warmed (37°C) humidified air.

STORM imaging buffer was freshly prepared before data acquisition, which contained 7 µl of oxygen-scavenging GLOX buffer (14 mg of glucose oxidase, 50 µl of 17 mg/mL catalase in 200 µl of 10 mM Tris, 50 mM NaCl, pH 8.0), 70 µl of MEA buffer (77 mg MEA in 1.0 mL 0.25N HCl) and 620µl of Buffer B (50 mM Tris-HCl (pH8.0) + 10 mM NaCl + 10% Glucose). For live-cell STORM imaging, it's 7 µl of oxygen-scavenging GLOX buffer, 3.5µl of BME, and 690µl phenol red-free media growth medium with 75 mM HEPES.

Förster resonance energy transfer, fluorescence resonance energy transfer (FRET)

FRET-based SHP2 biosensor (Addgene) was transfected in control and SENP8 KO HEK293T cells. After 48 h of transfection, the cells were hungered and stimulated with EGF (10ng/ml) or not and fixed by 4% PFA. Acceptor photobleaching FRET was performed using the FV1000 confocal microscope (Olympus). The ECFP and Ypet fluorophores were excited with excitation wavelengths of 405 and 514 nm, respectively. After acquiring the emission images, the cells were marked by a region of interest, and this region was bleached by a high laser power (20 iterations, 100% laser power, 514 nm). The FRET efficiency was measured as the percentage increase of donor after photobleaching the acceptor: FRET/ECFP ratio = 100% × (ECFPpost − ECFPpre)/ECFPpost. Spectral FRET was performed using the FV3000 confocal microscope (Olympus). The spectrum was measured from 450 nm to 580 nm in 10 nm

increments with excitation from 405 nm. The maximum fluorescence emission intensity of ECFP and Ypet fluorophores was 460nm and 520 nm. FRET ratio = 100% ×fluorescence Spectra λ_{520} / fluorescence Spectra λ_{460}.

***In vitro* SHP2 binding and activation assays**

In SHP2 binding assays, beads bounded wild-type or 4YF mutant SIRPα proteins were purified from HEK293T and incubated with recombinant SRC in reaction buffer (20 mM HEPES, pH 7.5, 150 mM NaCl, 10 mM $MgCl_2$, 5 mM ATP, and 10 mM BME) at 37°C for 30 min. Thereafter, these beads with bound proteins were washed five times in washing buffer PBS-T (PBS + 0.1% Tween 20). Subsequently, phosphorylated SIRPα was incubated with recombinant SHP2 at 37°C for 1 h. The beads were then washed 5 times with washing buffer and finally eluted with loading buffer for western blot analysis. For SHP2 allosteric inhibition, recombinant SHP2 was incubated with SHP099 before binding with SIRPα. In SHP2 activation assays, phosphorylated SIRPα was incubated with recombinant SHP2 or SHP2 E76K at 37°C for 1 h. Then, substrate DiFMUP was added and reacted at 25 °C for 30min. The reaction was then quenched, and the fluorescence signal was monitored using excitation and emission wavelengths of 340nm and 450nm on a microplate reader (M5, Molecular Devices).

Fluorescent labeling

Proteins, as well as antibodies, were labeled using AlexaFluor488 NHS Ester (Succinimidyl Ester, Thermo Fisher Scientific) reconstituted in anhydrous DMSO (dimethylsulfoxide, Sigma Aldrich). Dye was mixed with protein at a 5X molar ratio (dye: protein ratio was 5:1) and incubated at room temperature for 1 h. Excess dye was

removed by purifying protein over NAP-5 columns (GE Healthcare). Labeling was confirmed using NanoDrop 2000 (Thermo Fisher).

Bead preparation

~8X10^5 streptavidin beads were washed three times with PBS, mixed within PBS plus 0.4% BSA, and incubated with biotinylated mouse CD47 or anti-streptavidin IgG at room temperature for 0.5 h with end-over-end mixing to allow for protein coupling. For the specific purpose, IgG and mouse CD47 were labeled using AlexaFluor488 NHS Ester. Beads were then washed 3 times to remove excess protein. Unless otherwise indicated, anti-streptavidin mouse IgG (BioLegend) was added at 10 nM as the lowest IgG concentration that triggered engulfment. Biotinylated mouse CD47 (ACRObiosystem) was added at 50 nM for target particle experiments.

Quantification of engulfment by High Content Screening

1X10^4 BMDMs were plated in one well of a 96-well Plate 24h before the experiment. To disrupt SHP1 and SHP2 function, inhibitors were added for 4h while Mn^{2+} mediated integrin activation lasted 30min. To disrupt integrin function, the blocking antibodies or isotype control were added at 10 mg/mL 30 min before beads as macrophages were washed into non-serum culture media before antibody treatment to eliminate any potential serum components that may serve as integrin ligands. ~8X10^5 beads were added to the well and engulfment was allowed to proceed for 30 min. The plate was washed 3 times to remove extra beads. Cells were fixed with 4% PFA and stained with CellMask (Invitrogen) and DAPI. Images were obtained with the Operetta high-content cell imaging analysis system (PerkinElmer). Data were analyzed by images collected

from 10 representative fields in each group. Single cells were identified based on DAPI as a reference, while the beads quantification was performed based on the area and intensity of the beads channel.

Subcellular Protein Fractionation Extraction

CD47 coating beads were added to BMDMs and interaction was allowed to proceed for 30 min. The BMDMs were washed 3 times to remove extra beads. Then use the subcellular Protein Fractionation Kit (Thermo Fisher) according to the manual.

GTPases activation assay

BMDMs were lysed and the supernatants were used to carry out a GST precipitation assay to detect the GTPases activation. GTP-bound RAP1 was assayed by binding to recombinant GST-fused RalGDS. RAP1 activation was normalized to total RAP1.

Bimolecular fluorescence complementation

The bimolecular fluorescence complementation (BiFC) system is established by fusing two complementary parts of green fluorescent protein (GFP), GFP S1-10 and GFP S11, SIRPαGFP S1-10. SHP2 WT and 2KR GFP S11 were transfected into HEK293T cells, after 48h, cells were added to CD47 coated chamber and then stained with Na/K ATPase antibody (Abcam) to visualize membrane.

Precipitation with integrin tail related proteins

BMDMs were washed and treated with 5 mM DTBP (Thermo Fisher Scientific) for 30 min, and then lysed with TBS lysis buffer (containing 1% Triton X-100, 0.05% NP-40, Protease Inhibitor Cocktail, 1 mM $Cacl_2$, 1 mM $Mgcl_2$) for 30 min on ice. The lysates were then immunoprecipitated with indicated antibodies. Before western blot,

crosslinks with DTBP need to be cleaved by reducing the disulfide bond of the spacer arm with 100mM DTT at 37°C for 30 minutes.

***In vivo* recover tumor cells assay**

MC38 cells were labeled with Cell Proliferation Dye eFluor450 (Invitrogen) or eFluor670(Invitrogen) for 15min at room temperature respectively. Directly after labeling, eFluor670 dyed cells were opsonized by isotype control (Bio X Cell) and eFluor450 dyed cells by anti-PDL1 antibody (Bio X Cell). Subsequently, *Senp8*$^{+/+}$ and *Senp8*$^{+/-}$ mice were injected intraperitoneally (i.p.) with 1×10^7 cells in 200μl PBS in a 1:1 mixture. At 24h after injection, mice were euthanized, the peritoneal cavity was washed with PBS containing 5mM EDTA, and the absolute recovered number of cells was determined by NovoCyte flow cytometry (Agilent).

Tumor cells phagocytosis and digestion Assay

BMDMs were plated 8X10^4 per well in a 24-well plate and were transfected with SHP2WT, SHP2 K358R/ K364R plasmid, or empty vector. MC38-hPDL1 cells were opsonized with a range of anti-hPDL1 antibody (BioLegend) concentrations and stained with Cell Proliferation Dye eFluor450 at 37°C for 15 min. Each phagocytosis reaction reported in this work was performed by co-culture of 2X10^5 target cells and macrophages for 1 h at 37°C. Macrophages were identified by FITC-labeled anti-CD11b (BioLegend) as flow cytometry (Agilent) was performed. Phagocytosis was calculated as the percentage of eFluor450$^+$ CD11b$^+$ cells among CD11b$^+$ cells. In the same way, endogenous PDL1 opsonized MC38 cells were co-cultured with BMDMs for phagocytosis reaction, then extra tumor cells were washed out. Tumor cell digestion

lasted 24h, Then BMDMs were detached from the plate and determined by NovoCyte flow cytometry (Agilent).

Histology and Immunohistochemistry

Haematoxylin-eosin (HE) staining and Masson's trichrome stain were performed according to established staining protocols of our routine laboratory. For immunohistochemistry (IHC) staining, IHC staining of human tissue and organoids was performed according to established staining protocols of the routine laboratory, and IHC staining of mice tumors was performed with Multi-fluorescent Immunohistochemical Staining Kit (Absin) according to the manufacturer's instructions.

Tumor cell phagocytosis imaging

For the 3D image, CSFE (Invitrogen) labeled MC38 cells were opsonized by anti-PDL1 antibody, and tumor cells were added to Lamp1-mcherry overexpressed BMDMs for 1 h at 37°C. Then extra tumor cells were washed out and cells were stained with DAPI. For live-cell imaging, PDL1 antibody opsonized MC38 cells were stained with Hoechst 33342 to show nucleus, BMDMs were stained with Lysotracker to show lysosomes, and cells were imaged every 0.5 min after 10min co-culture. Cells were imaged on a heated stage and supplemented with warmed (37°C) humidified air. Fluorescent images were processed and assembled into figures using Fiji. Z-stack was created and rendered with Imaris (Bitplane).

ELISA

Tumor tissues were minced and 50–100 mg tissues were homogenized in NP40

(w:v = 1:5). Tumor homogenates were centrifuged at 12,000g for 30 min, then the supernatants were filtered through a 0.22 μm filter. Cytokine levels in tissue homogenates were measured by the mouse ELISA kit (Invitrogen) according to the manufacturer's instructions. In the same way, human blood plasma was utilized to detect human CEA. Absorbance at 450 nm was measured on a microplate reader (M5, Molecular Devices).

Deneddylation assay

For the deneddylation assay in macrophage extracts, the fluorogenic substrate, NEDD8-7-amino-4-methylcoumarin (R&D Systems) was used according to the instructions provided by the manufacturer. For tumor cell and macrophage interaction, MC38 cells were pre-incubated with pep-20-D12 (awsATWSNYwrh, lowercase letters in the sequence mean D-configuration amino acids) for CD47/SIRPα blockage, before adding to macrophages for co-culture for 30min at 37°C. Macrophages were sorted out by CD68 conjugated beads and then lysed in RIPA buffer with normalized protein concertation. For ALI-PDOs, organoid cultures were established as above and supplemented with 10μg /ml anti-CD47(BIO x cell) antibody or control for 7 days, then dissociated in collagenase IV. The single-cell suspension is derived from 0.05% trypsin/EDTA digestion for 5 min at 37 °C. Infiltrating macrophages were sorted out by CD68 conjugated beads, lysed in RIPA buffer and protein concentration was normalized. In the same way, to analyze the deneddylation activity of human peripheral blood leukocytes, cells were lysed in RIPA buffer and protein concentration was normalized.

Molecular docking and molecular dynamics

Chemically conjugated docking application from Rosetta program suite version 3.4 was used to dock NEDD8 to SHP2. Cryo-EM structure models of SHP2 (PDB ID 5ehr) were first relaxed by using the Relax ScriptManager application and models with the lowest energy scores were chosen for Chemically conjugated docking of NEDD8. There are two important novel chunks of code associated with this algorithm. The C-terminus of NEDD8 (glycine) is chemically linked to K364 of SHP2, resulting in an isopeptide bond between the proteins. It is this bond that this protocol remodels. Phil Bradley deserves credit for helping set up this chemical conjugation code. The remodeling algorithm is straightforward. It uses Rosetta's standard Metropolis/Monte Carlo random sampling tools. A series of possible Pose modifications are chosen from each Monte Carlo cycle. These include effective psi and phi angles of NEDD8's terminal glycine. These are treated directly by TorsionDOFMover instead of more familiar sidechain/backbone movers because the extra chemical bond changes the torsional preferences at these bonds, meaning that the Ramachandran and Dunbrack libraries do not apply. TorsionDOFMover internally checks against a molecular-mechanics bond torsion term (although this term is not in the broader score function). Other possible Monte Carlo moves include standard Small/Shear moves on the penultimate NEDD8 residues (the number of mobile residues is command-line flagged), and also KIC loop modeling. After a random move, the pose runs through Rotamer Trials (to quickly pack sidechains) and a minimization step before the Metropolis criterion is applied. Some fraction of MC cycles instead performs a full repack of the interface. All the molecular graphics were rendered by the UCSF Chimera57 software

version. Molecular dynamics (MD) simulations were conducted by GROMACS.

Microscale thermophoresis

The interaction between 2P-IRS-1 and NEDD8-SHP2 conjugation was measured using the Monolith NT.115 MST instrument (Nanotemper Technologies). 2P-IRS-1 were fluorescently labeled with Cy5. The NEDD8-SHP2 conjugation conducted from *in vitro* neddylation assay was purified by Superdex 75 gel filtration column (GE Healthcare) and the purities were determined by SDS-PAGE. A solution of unlabeled protein was serially diluted in the presence of 100 nM labeled 2P-IRS-1. The samples were loaded into capillaries (Nanotemper Technologies). Measurements were performed at 25 °C, Data analyses were performed using Nanotemper Analysis software.

Real-time PCR

Total RNA of human peripheral blood leukocytes was extracted by Trizol and then reverse-transcribed into cDNA using ReverTraAce qPCR RT kit (Toyobo). Real-time PCR was conducted using an SYBR Green reagent (CWBIO) on CFX96 Touch Real-Time PCR Detection System (Bio-Rad). Primer sequences are listed in the Supplemental Table 1.

RNA sequencing

A total amount of 4 μg RNA/sample was used for the RNA sample preparations. Sequencing libraries were generated using the NEBNext® UltraTM RNA Library Prep Kit for Illumina® (NEB, USA) and index codes were added to attribute sequences to each sample. Library preparation, clustering and sequencing were done by Novogene Experimental Department (Novogene). The GEO number of RNA-seq is GSE199585.

Mass spectrometry

HEK293T lysates were separated on SDS-PADE and followed by in-gel digestion, desalted, and then analyzed with the assistance of PTM-Biolabs Inc. Data analysis was carried out with Maxquant (v1.6.8.0).

Proximity ligation assay

Duolink® Proximity Ligation Assay (Sigma-Aldrich) was conducted according to the manual. PLA technology uses a pair of monoclonal or polyclonal antibody probes labeled with an oligodeoxynucleotide (single-stranded DNA). When the two probes recognize the same protein, the distance between the two probes is close, resulting in the so-called proximity effect (proximity). The fragmented DNA on the PLA probe can be joined together to form a new DNA fragment by ligase. The new DNA fragment can be amplified and quantified by fluorescent PCR, so as to quantify the corresponding target protein.

Cytometry by Time-Of-Flight (CyTOF)

The tumor was minced and digested in DMEM containing 300 units /mL collagenase IV (Worthington) and 50 U/ml DNase I (Sigma) at 37°C for 1 h. Red blood cells are removed by ACK Lysing Buffer. The cell mixtures were then filtered through 75 mm cell strainers. For mass cytometry analysis, purified antibodies panel were listed in Supplemental Table 4. Antibody labeling with the indicated metal tag was performed using the MaxPAR antibody Labelling kit (Fluidigm). Conjugated antibodies were titrated for optimal concentration before use. Cells were thawed and washed with PBS and then stained with 100μL of 250nM cisplatin (Fluidigm) for 5min on ice to exclude

dead cells, and then incubated in Fc receptor blocking solution before stained with surface antibodies cocktail for 30 min on ice. Cells were washed twice with FACS buffer (PBS+0.5%BSA) and fixed in 200μL of intercalation solution (Maxpar Fix and Perm Buffer containing 250nM 191/193Ir, Fluidigm) overnight. After fixation, cells were washed once with FACS buffer and then perm buffer (eBioscience), stained with intracellular antibodies cocktail for 30 min on ice. Cells were washed and resuspended with deionized water, adding into 20% EQ beads (Fluidigm), acquired on a mass cytometer (Helios, Fluidigm). Data analysis was done by PLTTECH Experimental Department (PLTTECH). The accession number of FlowRepository is FR-FCM-Z563.

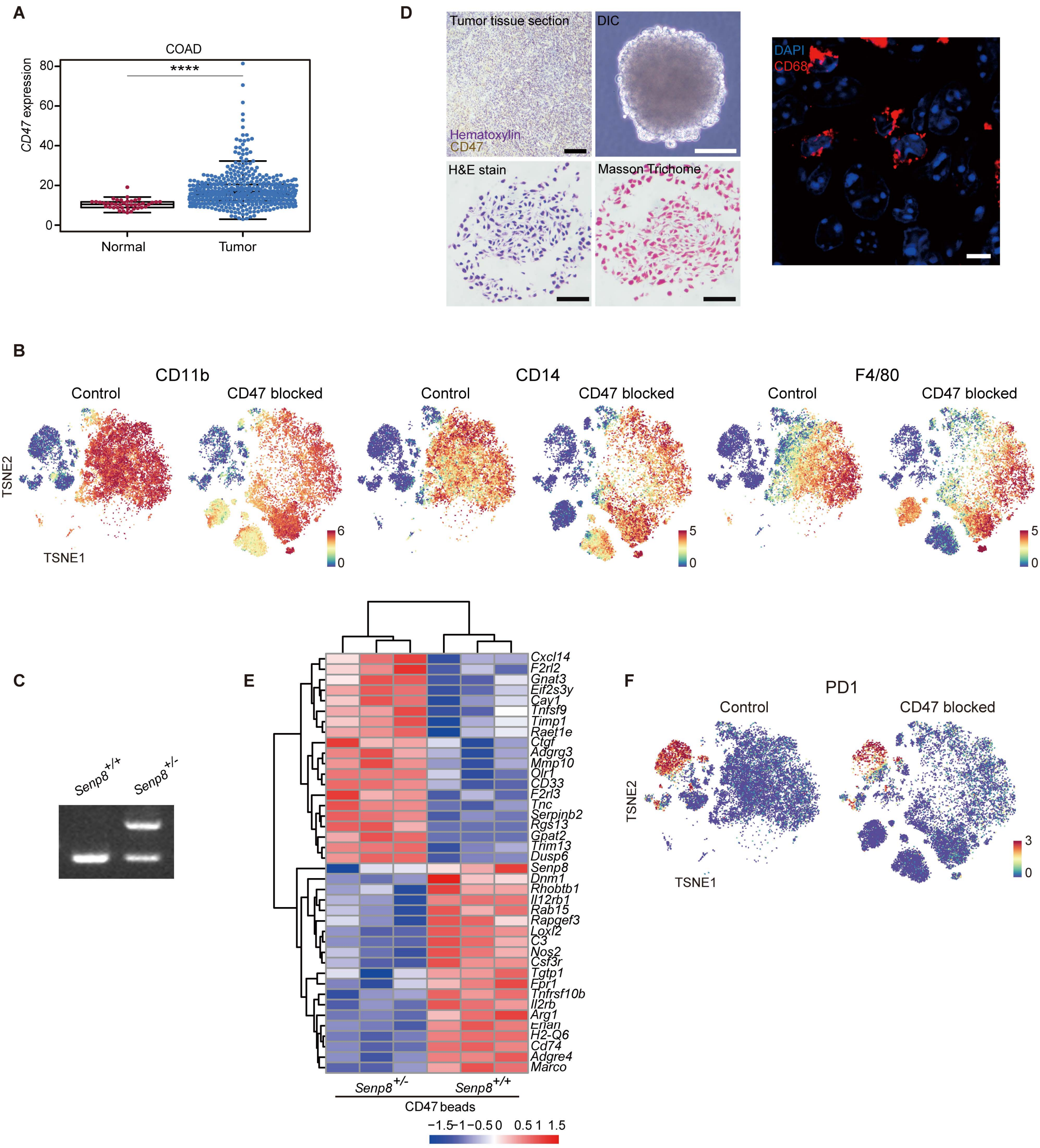
A
COAD
CD47 expression
****
80
60
40
20
0
Normal
Tumor
D
Tumor tissue section
Hematoxylin
CD47
DIC
H&E stain
Masson Trichome
DAPI
CD68
B
CD11b
CD14
F4/80
Control
CD47 blocked
TSNE2
TSNE1
6
5
0
C
Senp8+/+
Senp8+/-
E
Cxcl14
F2rl2
Gnat3
Eif2s3y
Cav1
Tnfsf9
Timp1
Raet1e
Ctgf
Adgrg3
Mmp10
Olr1
CD33
F2rl3
Tnc
Serpinb2
Rgs13
Gpat2
Trim13
Dusp6
Senp8
Dnm1
Rhobtb1
Il12rb1
Rab15
Rapgef3
Loxl2
C3
Nos2
Csf3r
Tgtp1
Fpr1
Tnfrsf10b
Il2rb
Arg1
H2-Q6
Cd74
Adgre4
Marco
Senp8+/-
Senp8+/+
CD47 beads
−1.5 −1 −0.5 0 0.5 1 1.5
F
PD1
Control
CD47 blocked
TSNE2
TSNE1
3
0

Supplemental Figure 1. CD47 blockage modulates neddylation of CRC TIMs.

(A) The *CD47* expression from TCGA-COAD cohort (n of adjacent normal colon tissue samples=41, n of colon adenocarcinoma tissue samples=473). (B) TSNE visualization of CD11b, CD14, and F4/80 expression in tumor-infiltrating leukocytes from Figure 1D groups. (C) Genotyping of *Senp8* heterozygous mice strain. (D) CD47 stain of human surgical tumor resections as well as DIC, H&E stain, and Masson's trichrome stain of organoids derived from human surgical tumor resections, scale bars, 100μm (left). Confocal microscopy visualization of organoid stained with CD68 and DAPI to show TIMs, scale bars, 10μm (right). (E) Heatmap of top genes differentially expressed in indicated BMDMs (n=3). (F) TSNE visualization of PD1 expression in tumor-infiltrating leukocytes from Figure 1D groups. Data are expressed as mean ± SD; Wilcoxon test (A); non-significant (ns), $p > 0.05$; $^{*}p < 0.05$; $^{**}p < 0.01$; $^{***}p < 0.001$; $^{****}p < 0.0001$.

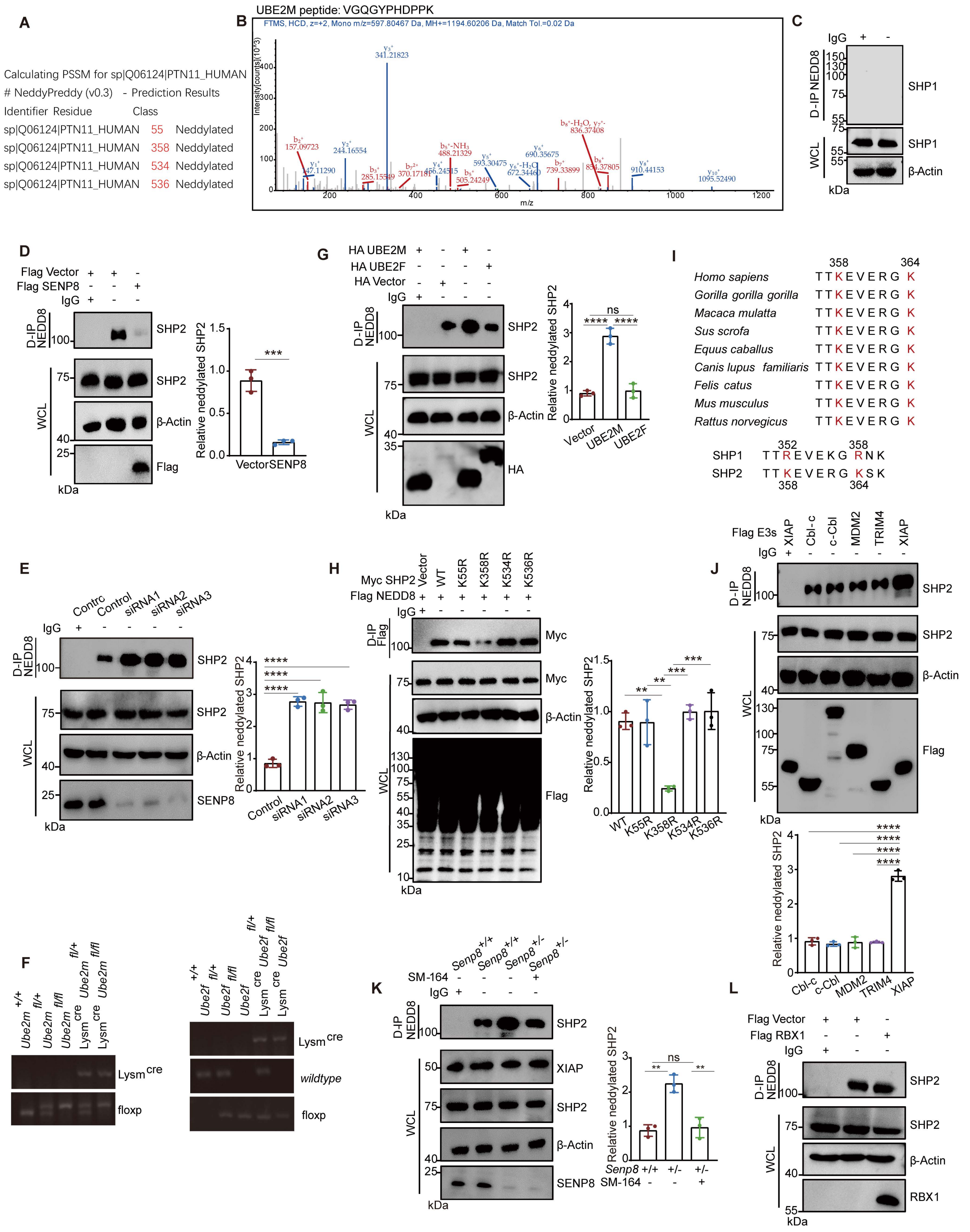
A
Calculating PSSM for sp|Q06124|PTN11_HUMAN
NeddyPreddy (v0.3) - Prediction Results
Identifier Residue Class
sp|Q06124|PTN11_HUMAN 55 Neddylated
sp|Q06124|PTN11_HUMAN 358 Neddylated
sp|Q06124|PTN11_HUMAN 534 Neddylated
sp|Q06124|PTN11_HUMAN 536 Neddylated
B
UBE2M peptide: VGQGYPHDPPK
FTMS, HCD, z=+2, Mono m/z=597.80467 Da, MH+=1194.60206 Da, Match Tol.=0.02 Da
Intensity[counts](10^3)
m/z
C
IgG
D-IP NEDD8
WCL
SHP1
β-Actin
kDa
D
Flag Vector
Flag SENP8
IgG
D-IP NEDD8
WCL
SHP2
β-Actin
Flag
kDa
Relative neddylated SHP2
Vector
SENP8
E
Control
siRNA1
siRNA2
siRNA3
IgG
D-IP NEDD8
WCL
SHP2
β-Actin
SENP8
kDa
Relative neddylated SHP2
F
Lysm cre
floxp
wildtype
G
HA UBE2M
HA UBE2F
HA Vector
IgG
D-IP NEDD8
WCL
SHP2
β-Actin
HA
kDa
Relative neddylated SHP2
Vector
UBE2M
UBE2F
ns
H
Myc SHP2
Vector
WT
K55R
K358R
K534R
K536R
Flag NEDD8
IgG
D-IP Flag
WCL
Myc
β-Actin
Flag
kDa
Relative neddylated SHP2
I
358
364
Homo sapiens TTKEVERGK
Gorilla gorilla gorilla TTKEVERGK
Macaca mulatta TTKEVERGK
Sus scrofa TTKEVERGK
Equus caballus TTKEVERGK
Canis lupus familiaris TTKEVERGK
Felis catus TTKEVERGK
Mus musculus TTKEVERGK
Rattus norvegicus TTKEVERGK
352
358
SHP1 TTREVEKGRNK
SHP2 TTKEVERGKSK
358
364
J
Flag E3s
XIAP
Cbl-c
c-Cbl
MDM2
TRIM4
XIAP
IgG
D-IP NEDD8
WCL
SHP2
β-Actin
Flag
kDa
Relative neddylated SHP2
K
Senp8 +/+
Senp8 +/+
Senp8 +/-
Senp8 +/-
SM-164
IgG
D-IP NEDD8
WCL
SHP2
XIAP
β-Actin
SENP8
kDa
Relative neddylated SHP2
Senp8
ns
L
Flag Vector
Flag RBX1
IgG
D-IP NEDD8
WCL
SHP2
β-Actin
RBX1
kDa

Supplemental Figure 2. Neddylation cascade of SHP2.

D-IP: Denaturing immunoprecipitated, WCL: the whole-cell lysate. (A) Potential neddylated lysine sites of SHP2 predicted by NeddyPreddy. (B) Mass spectrum of the UBE2M peptide from SHP2 co-immunoprecipitate in lysates of HEK293T cells. (C) Western blot of BMDMs indicating that neddylation is not involved in SHP1. D-E SENP8 deneddylated SHP2 (n=3). HEK293T cells were transfected with SENP8 siRNA (D) or over-expressed with SENP8-Flag (E). (F) Genotyping of macrophage-specific *Ube2f* $^{m\varphi-/-}$ and *Ube2m*$^{m\varphi-/-}$ mice strain. (G) UBE2M mediated SHP2 neddylation in HEK293T cells (n=3). (H) SHP2 mutation K358R did not completely abolish its neddylation in HEK293T cells (n=3). (I) Sequence alignment of the region which contains the neddylation site of SHP2 in different species (up). Sequence alignment between SHP1 and SHP2 (down). J-K XIAP mediated SHP2 neddylation in HEK293T cells and BMDMs (n=3). SM-164: inhibitor of XIAP (100 nM,4h). (L) RBX1 (neddylation E3 of Cullin1) RBX1 was not involved in SHP2 neddylation in HEK293T cells. Data are expressed as mean ± SD; 2-tailed unpaired Student's t-test (D), 1-way ANOVA followed by Tukey's posthoc test (E, G, H, J, K); non-significant (ns), $p > 0.05$; $^{*}p < 0.05$; $^{**}p < 0.01$; $^{***}p < 0.001$; $^{****}p < 0.0001$.

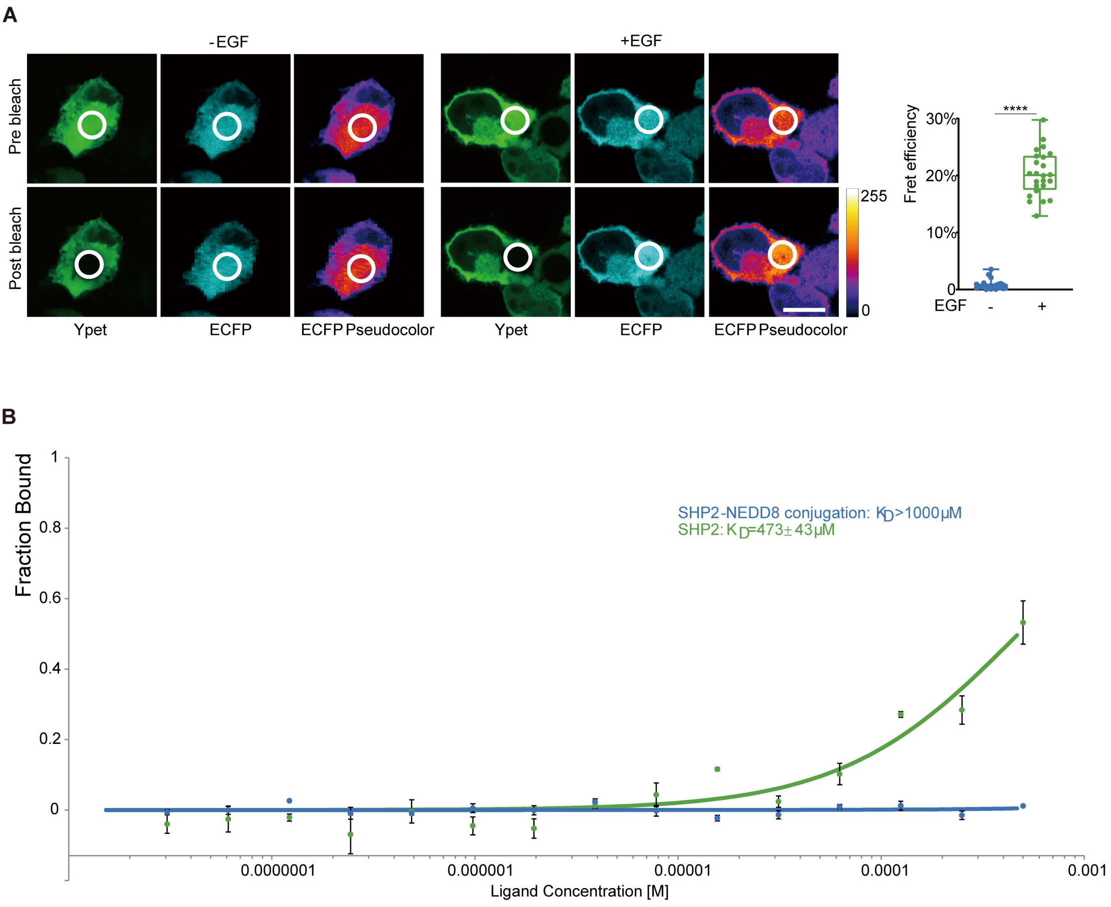
A
-EGF
+EGF
Pre bleach
Post bleach
Ypet
ECFP
ECFP Pseudocolor
Ypet
ECFP
ECFP Pseudocolor
255
0
Fret efficiency
****
30%
20%
10%
0
EGF
-
+
B
Fraction Bound
1
0.8
0.6
0.4
0.2
0
SHP2-NEDD8 conjugation: KD>1000μM
SHP2: KD=473±43μM
0.0000001
0.000001
0.00001
0.0001
0.001
Ligand Concentration [M]

Supplemental Figure 3. SHP2 neddylation inhibits its binding to phospho-ligand. (A) FRET efficiency of SHP2 biosensor was assessed by acceptor photobleaching EGF stimulation (10ng/ml,15min), scale bars, 10μm, (cell number=24). (B) MST measurement of SHP2 and NEDD8-SHP2 conjugation binding to fluorescently labeled 2P-IRS-1, (n=3). Data are expressed as mean ± SD; 2-tailed unpaired Student's t-test (A); non-significant (ns), $p > 0.05$; $^{*}p < 0.05$; $^{**}p < 0.01$; $^{***}p < 0.001$; $^{****}p < 0.0001$.

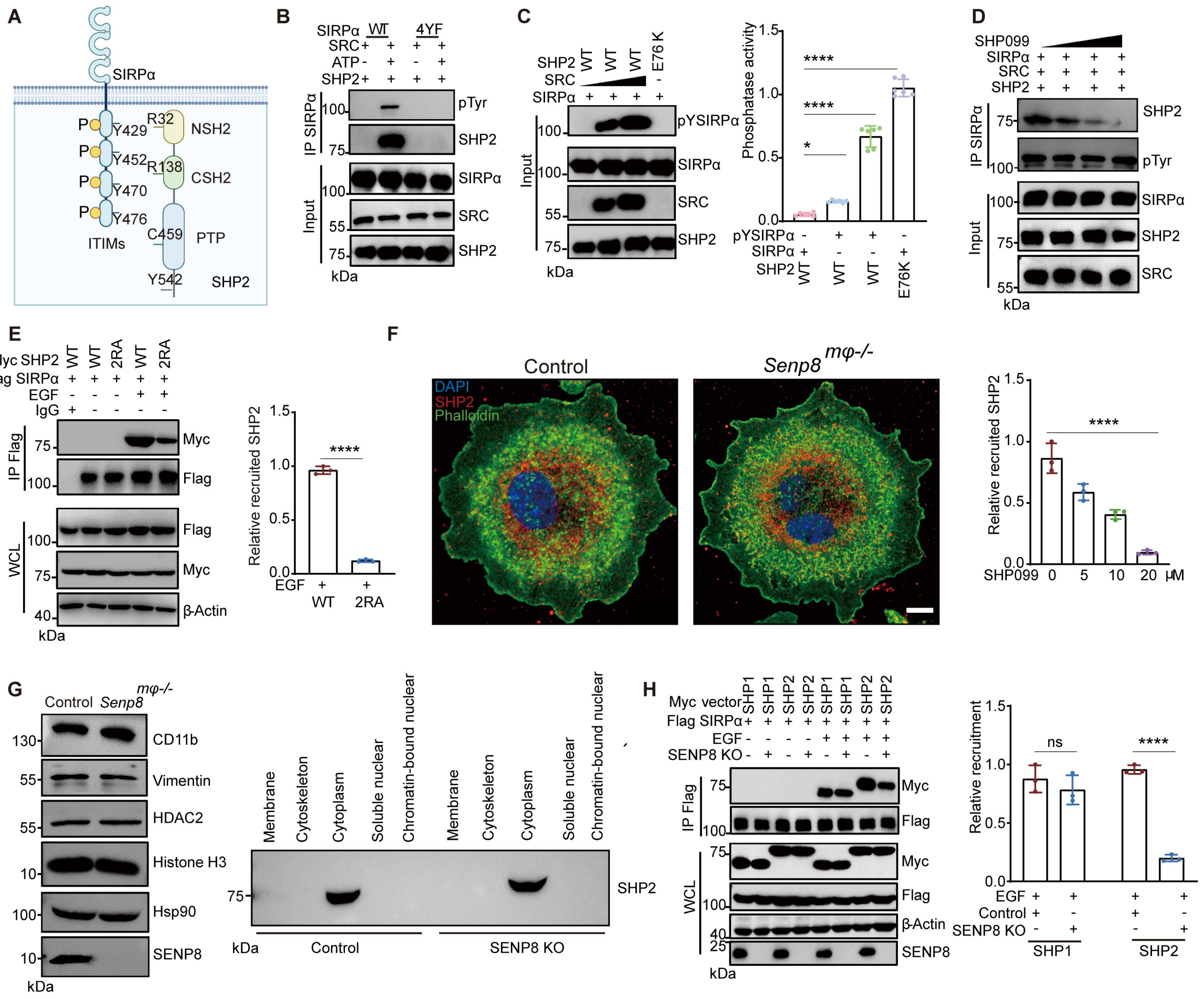
A
SIRPα
Y429
Y452
Y470
Y476
ITIMs
R32
NSH2
R138
CSH2
C459
PTP
Y542
SHP2
B
SIRPα WT 4YF
SRC
ATP
SHP2
IP SIRPα
Input
pTyr
SHP2
SIRPα
SRC
kDa
C
SHP2 WT WT WT E76 K
SRC
SIRPα
pYSIRPα
Input
Phosphatase activity
pYSIRPα
D
SHP099
IP SIRPα
pTyr
Input
E
Myc SHP2 WT WT 2RA WT 2RA
Flag SIRPα
EGF
IgG
IP Flag
Myc
Flag
WCL
β-Actin
Relative recruited SHP2
F
Control
Senp8 mφ-/-
DAPI
SHP2
Phalloidin
SHP099 0 5 10 20 μM
G
CD11b
Vimentin
HDAC2
Histone H3
Hsp90
SENP8
Membrane
Cytoskeleton
Cytoplasm
Soluble nuclear
Chromatin-bound nuclear
Control
SENP8 KO
H
Myc vector SHP1 SHP1 SHP2 SHP2 SHP1 SHP1 SHP2 SHP2
SENP8 KO
Relative recruitment
ns
SHP1
SHP2

Supplemental Figure 4. Phosphorylated SIRPα recruits and activates SHP2.

D-IP: Denaturing immunoprecipitated, WCL: the whole-cell lysate. (A) The schematic diagram of the SIRPα ITIM motif activating SHP2 was shown. (B) SIRPα and its mutation protein were phosphorylated by SRC *in vitro,* and SHP2 was added to detect its binding. (C) SIRPα proteins were phosphorylated by SRC *in vitro*. Then SHP2 was mixed with pY-SIRPα, and *in vitro* SHP2 phosphatase assay showed phospho-ITIMs activated SHP2 (n=6). (D) SIRPα proteins were phosphorylated by SRC *in vitro*. SHP099 pre-treated SHP2 was mixed with pY-SIRPα to detect its binding (n=3). (E) Western blot indicating SHP2 recruitment toward SIRPα receptor under EGF stimulation (10ng/ml,15min) in HEK293T cells (n=3). (F) Confocal microscopy visualization of SHP2 localization in indicated BMDMs, scale bars, 10μm. (G) Western blot indicating SHP2 location of different subcellular fractionation in indicated BMDMs. (H) Western blot indicating that SHP2 but not SHP1 recruitment of SIRPα receptor was disrupted by neddylation under EGF stimulation (10ng/ml,15min) in HEK293T cells (n=3). Data are expressed as mean ± SD; 2-tailed unpaired Student's t-test (E), 1-way ANOVA followed by Tukey's posthoc test (C, D), 2-way ANOVA followed by Tukey's posthoc test H; non-significant (ns), $p > 0.05$; $^{*}p < 0.05$; $^{**}p < 0.01$; $^{***}p < 0.001$; $^{****}p < 0.0001$.

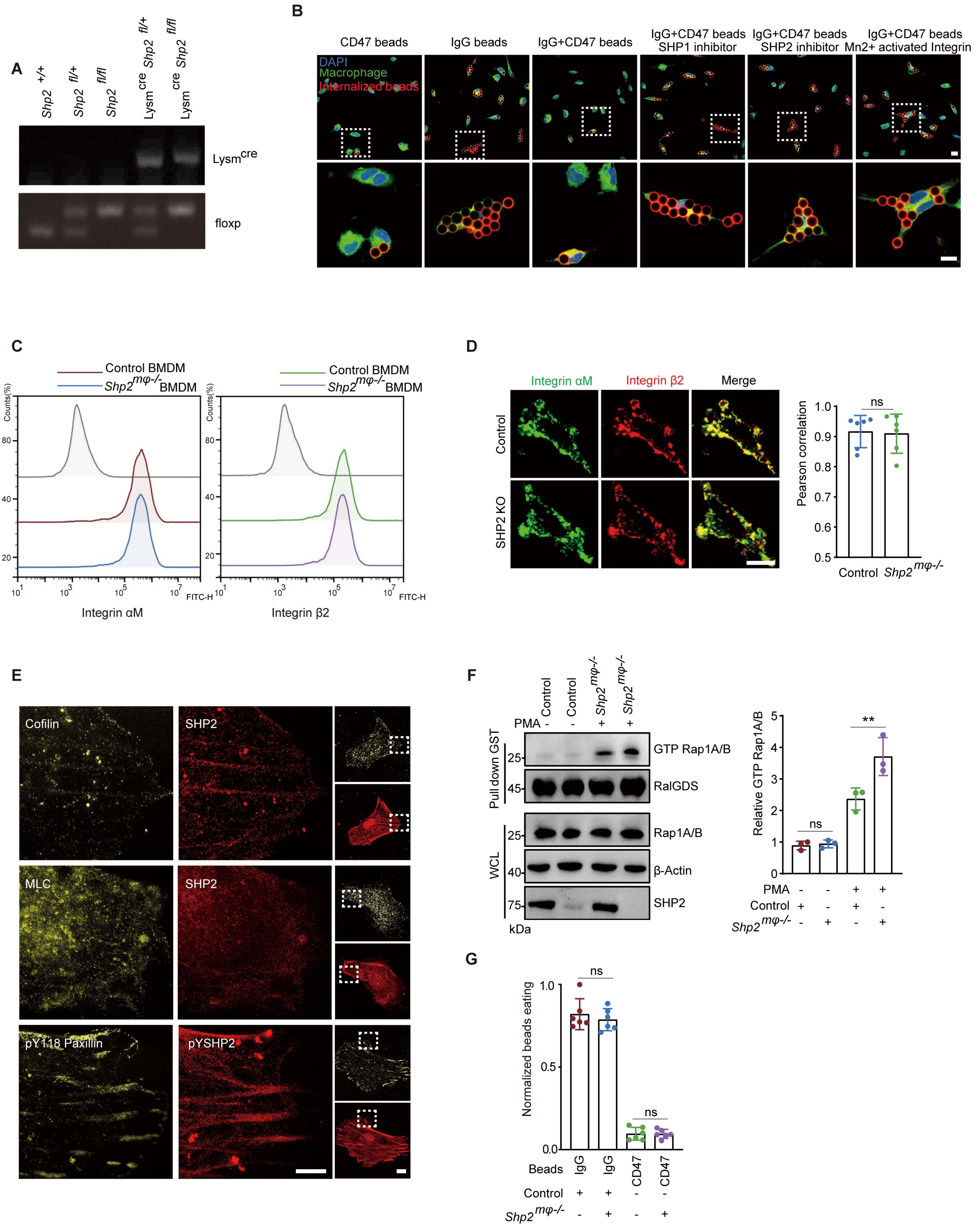

A
Shp2 +/+
Shp2 fl/+
Shp2 fl/fl
Lysm cre Shp2 fl/+
Lysm cre Shp2 fl/fl
Lysmcre
floxp
B
CD47 beads
IgG beads
IgG+CD47 beads
IgG+CD47 beads SHP1 inhibitor
IgG+CD47 beads SHP2 inhibitor
IgG+CD47 beads Mn2+ activated Integrin
DAPI
Macrophage
Internalized beads
C
Control BMDM
Shp2mφ-/- BMDM
Counts(%)
Integrin αM
Integrin β2
FITC-H
D
Integrin αM
Integrin β2
Merge
Control
SHP2 KO
Pearson correlation
ns
Control
Shp2mφ-/-
E
Cofilin
SHP2
MLC
SHP2
pY118 Paxillin
pYSHP2
F
PMA
Control
Shp2mφ-/-
Pull down GST
WCL
GTP Rap1A/B
RalGDS
Rap1A/B
β-Actin
SHP2
kDa
Relative GTP Rap1A/B
ns
**
G
Normalized beads eating
ns
Beads
IgG
CD47
Control
Shp2mφ-/-

Supplemental Figure 5. SHP2 mediation of the CD47/SIRPα axis relies on integrin αMβ2.

(A) Genotyping of macrophage-specific *Shp2*$^{m\varphi-/-}$ mice strain. (B) High content screening images depicted the normalized beads eating of indicated BMDMs described in Figure 6A. BMDMs were labeled with CellMask and DAPI, beads are outlined in red, scale bars, 10μm. (C) Surface expression of integrin αM or β2 in Control and SHP2 KO BMDMs were analyzed by flow cytometry. (D) Confocal microscopy images showed colocalization of integrin αM and β2 in indicated BMDMs (n=6), scale bars, 10μm. (E) The colocalization between SHP2 and Cofilin, Myosin / pY542 SHP2 and pY118 Paxillin were detected in MEF cells under PMA stimulation, scale bars, 5μm. (F) Western blot analysis of SHP2 deletion effect on Rap GTPase under PMA stimulation (100 ng/ml,15min) (n=3). (G) The normalized IgG or CD47 beads eating of indicated BMDMs (n=6). Data are expressed as mean ± SD; 1-way ANOVA followed by Tukey's posthoc test (G, F), Pearson correlation (D); non-significant (ns), $p > 0.05$; $^{*}p < 0.05$; $^{**}p < 0.01$; $^{***}p < 0.001$; $^{****}p < 0.0001$.

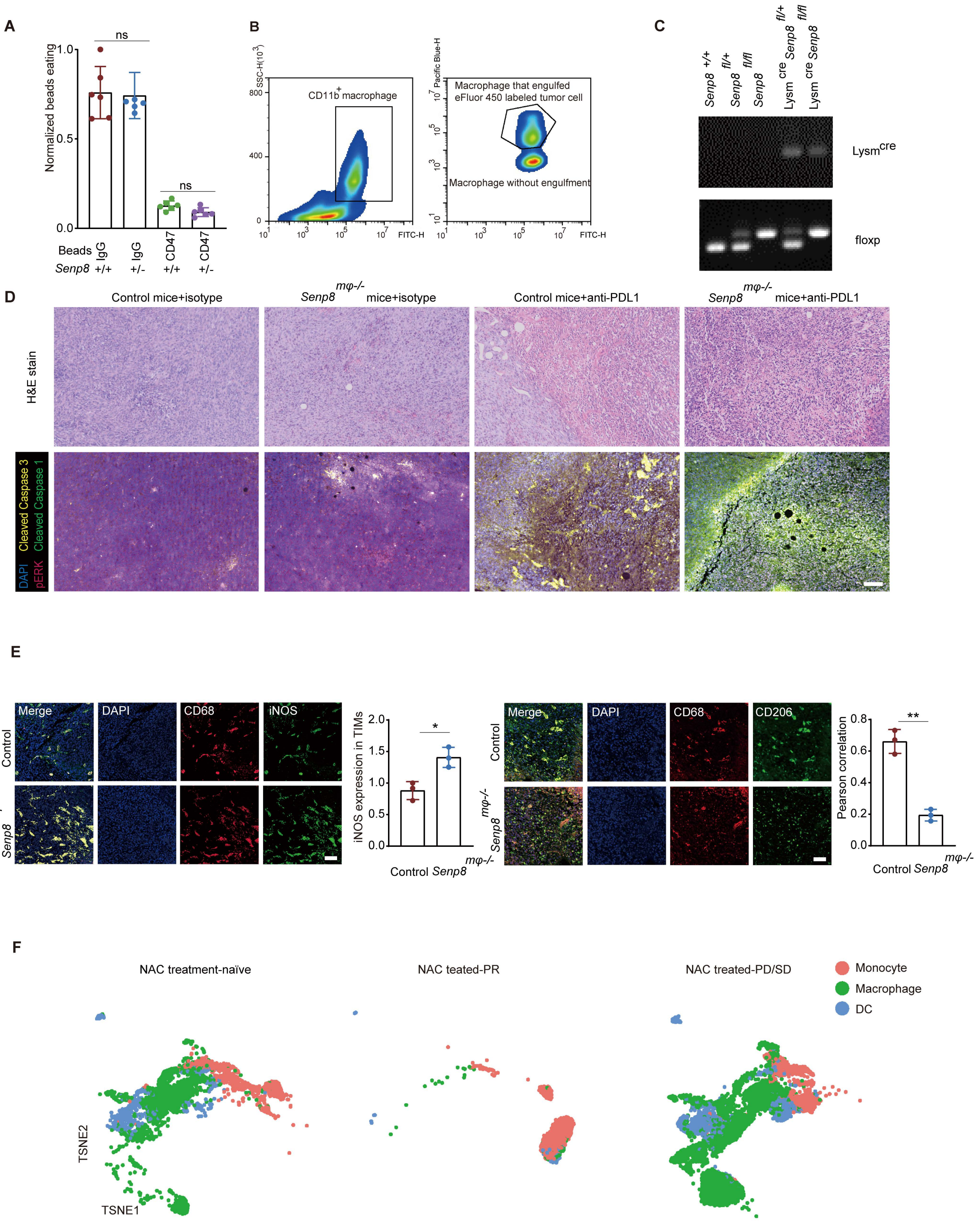
A
ns
Normalized beads eating
1.0
0.5
0.0
ns
Beads IgG IgG CD47 CD47
Senp8 +/+ +/- +/+ +/-
B
SSC-H(10^3)
800
400
0
CD11b+ macrophage
FITC-H
Pacific Blue-H
Macrophage that engulfed eFluor 450 labeled tumor cell
Macrophage without engulfment
FITC-H
C
Senp8 +/+
Senp8 fl/+
Senp8 fl/fl
Lysm cre Senp8 fl/+
Lysm cre Senp8 fl/fl
Lysmcre
floxp
D
Control mice+isotype
Senp8 mφ-/- mice+isotype
Control mice+anti-PDL1
Senp8 mφ-/- mice+anti-PDL1
H&E stain
DAPI pERK Cleaved Caspase 3 Cleaved Caspase 1
E
Merge DAPI CD68 iNOS
Control
Senp8 mφ-/-
iNOS expression in TIMs
2.0
1.5
1.0
0.5
0.0
*
Control Senp8 mφ-/-
Merge DAPI CD68 CD206
Control
Senp8 mφ-/-
Pearson correlation
0.8
0.6
0.4
0.2
0.0
**
Control Senp8 mφ-/-
F
NAC treatment-naïve
NAC teated-PR
NAC treated-PD/SD
Monocyte
Macrophage
DC
TSNE2
TSNE1

Supplemental Figure 6. Enhancement of SHP2 neddylation promotes macrophage engulfment in response to antibodies.

(A) The normalized beads eating of indicated BMDMs (n=6). (B) Representative flow analysis plots of BMDMs that had swallowed the tumor cells. Phagocytosis was calculated as the percentage of eFluor405^{+}CD11b^{+} cells among CD11b^{+} cells. (C) Genotyping of macrophage-specific *Senp8*$^{m\varphi-/-}$ mice strain. (D) Representative images of MC38 tumor section under 10X magnification of Figure 9E, scale bars, 100μm. (E) Images of indicated MC38 tumors (Figure 9E) staining iNOS, CD206 and CD68 (n=3), scale bars, 50μm. (F) TSNEs of tumor-infiltrating myeloid cells in all samples (n of treatment naïve samples=12, n of NAC-treated PR samples=8, and n of NAC-treated PD/SD samples=5). Data are expressed as mean ± SD; 1-way ANOVA followed by Tukey's posthoc test (A); Pearson correlation (E); 2-tailed unpaired Student's t-test (E). non-significant (ns), $p > 0.05$; $^{*}p < 0.05$; $^{**}p < 0.01$; $^{***}p < 0.001$; $^{****}p < 0.0001$.

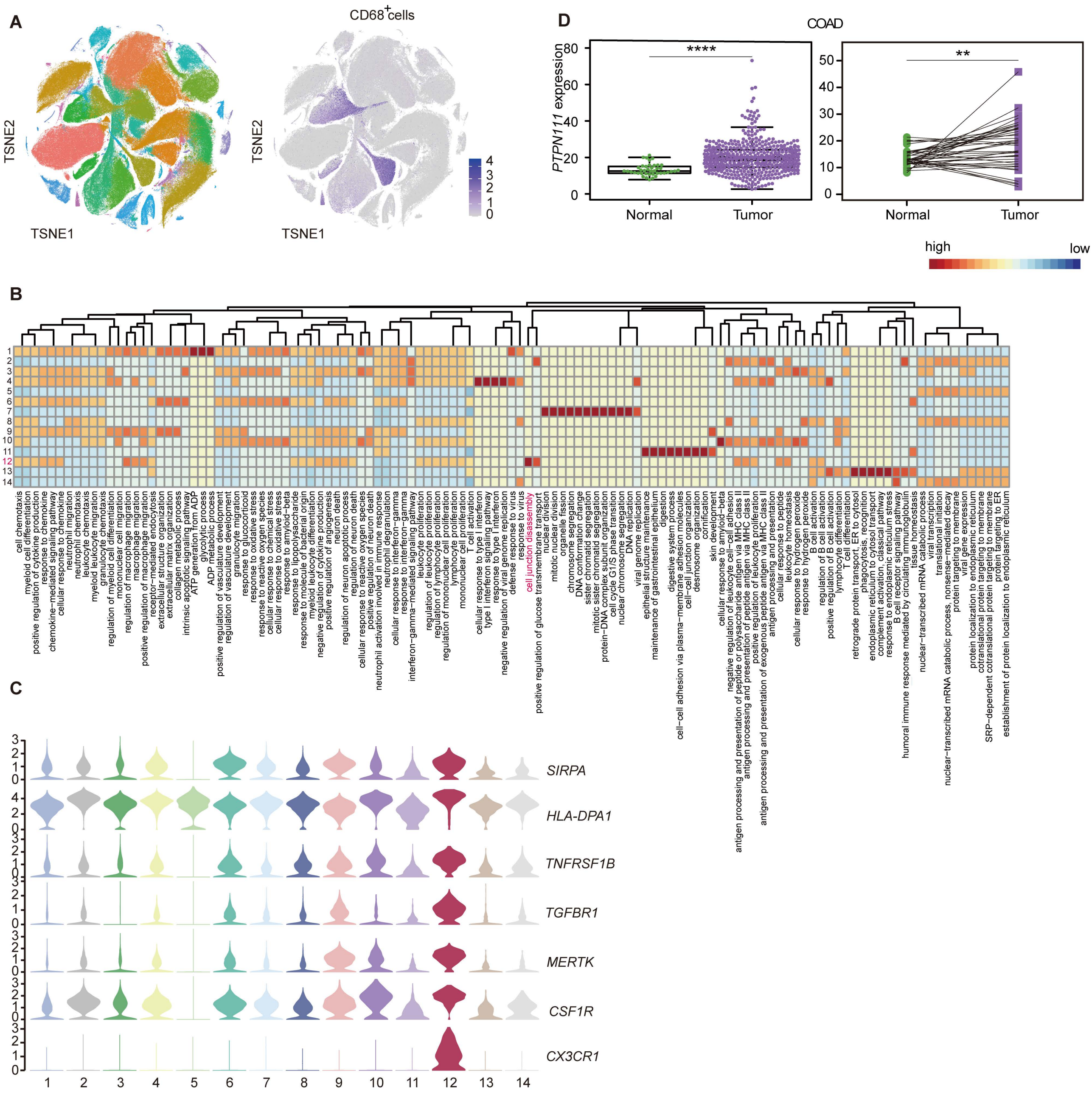

A
CD68+cells
TSNE2
TSNE1
TSNE2
TSNE1
4
3
2
1
0
D
COAD
PTPN111 expression
80
60
40
20
0
****
50
40
30
20
10
0
**
Normal
Tumor
Normal
Tumor
high
low
B
1
2
3
4
5
6
7
8
9
10
11
12
13
14
cell chemotaxis
myeloid cell differentiation
positive regulation of cytokine production
response to chemokine
chemokine−mediated signaling pathway
cellular response to chemokine
neutrophil migration
neutrophil chemotaxis
leukocyte chemotaxis
myeloid leukocyte migration
granulocyte chemotaxis
regulation of myeloid cell differentiation
mononuclear cell migration
regulation of macrophage migration
macrophage migration
positive regulation of macrophage migration
receptor−mediated endocytosis
extracellular structure organization
extracellular matrix organization
collagen metabolic process
intrinsic apoptotic signaling pathway
ATP generation from ADP
glycolytic process
ADP metabolic process
positive regulation of vasculature development
regulation of vasculature development
granulocyte migration
response to glucocorticoid
response to oxidative stress
response to reactive oxygen species
cellular response to chemical stress
cellular response to oxidative stress
response to amyloid−beta
response to lipopolysaccharide
response to molecule of bacterial origin
myeloid leukocyte differentiation
negative regulation of cytokine production
positive regulation of angiogenesis
neuron death
regulation of neuron apoptotic process
regulation of neuron death
cellular response to reactive oxygen species
positive regulation of neuron death
neutrophil activation involved in immune response
neutrophil degranulation
cellular response to interferon−gamma
response to interferon−gamma
interferon−gamma−mediated signaling pathway
leukocyte proliferation
regulation of leukocyte proliferation
regulation of lymphocyte proliferation
regulation of mononuclear cell proliferation
lymphocyte proliferation
mononuclear cell proliferation
T cell activation
cellular response to type I interferon
type I interferon signaling pathway
response to type I interferon
negative regulation of viral genome replication
defense response to virus
response to virus
cell junction disassembly
positive regulation of glucose transmembrane transport
nuclear division
mitotic nuclear division
organelle fission
chromosome segregation
DNA conformation change
sister chromatid segregation
mitotic sister chromatid segregation
cell cycle G1/S phase transition
protein−DNA complex subunit organization
nuclear chromosome segregation
DNA replication
viral genome replication
epithelial structure maintenance
maintenance of gastrointestinal epithelium
digestion
digestive system process
cell−cell adhesion via plasma−membrane adhesion molecules
cell−cell junction organization
desmosome organization
cornification
skin development
cellular response to amyloid−beta
negative regulation of leukocyte cell−cell adhesion
antigen processing and presentation of peptide or polysaccharide antigen via MHC class II
antigen processing and presentation of peptide antigen via MHC class II
positive regulation of leukocyte proliferation
antigen processing and presentation of exogenous peptide antigen via MHC class II
antigen processing and presentation
cellular response to peptide
leukocyte homeostasis
cellular response to hydrogen peroxide
response to hydrogen peroxide
B cell activation
regulation of B cell activation
positive regulation of B cell activation
lymphocyte differentiation
T cell differentiation
retrograde protein transport, ER to cytosol
phagocytosis, recognition
endoplasmic reticulum to cytosol transport
complement activation, classical pathway
response to endoplasmic reticulum stress
B cell receptor signaling pathway
humoral immune response mediated by circulating immunoglobulin
tissue homeostasis
nuclear−transcribed mRNA catabolic process
viral transcription
translational initiation
nuclear−transcribed mRNA catabolic process, nonsense−mediated decay
protein targeting to membrane
viral gene expression
protein localization to endoplasmic reticulum
cotranslational protein targeting to membrane
SRP−dependent cotranslational protein targeting to membrane
protein targeting to ER
establishment of protein localization to endoplasmic reticulum
C
SIRPA
HLA-DPA1
TNFRSF1B
TGFBR1
MERTK
CSF1R
CX3CR1
1
2
3
4
5
6
7
8
9
10
11
12
13
14

Supplemental Figure 7. The immunosuppressive microenvironment of colorectal cancer relies on the CD47/SIRPα axis.

(A) TSNEs of all type cells of dissociated CRC tumor tissues, $CD68^+$ cells were extracted (n of MMRd samples=28, n of MMRp samples=34). (B) GO analysis of tumor-infiltrating macrophage clusters in MMRd and MMRp tumor samples. (C) Violin plot showed the relative expression of cell clusters-associated genes in MMRd and MMRp tumor samples. (D)The *PTPN11* expression from TCGA-COAD cohort (n of adjacent normal colon tissue samples=41, n of colon adenocarcinoma tissue samples=473). Data are expressed as mean ± SD; Wilcoxon test (D); non-significant (ns), $p > 0.05$; $^{*}p < 0.05$; $^{**}p < 0.01$; $^{***}p < 0.001$; $^{****}p < 0.0001$.

Supplemental Table 1		
REAGENT or RESOURCE	SOURCE	IDENTIFIER
**Antibodies (applications)**		
Rabbit Anti CD47	Abcam	Cat: ab218810
Rabbit Anti SIRP alpha	Abcam	Cat: ab191419
Rabbit Anti SHP2 (phospho Y542)	Abcam	Cat: ab62322
Rabbit Anti NEDD8	Abcam	Cat: ab81264
Rabbit Anti UBE2M	Abcam	Cat: ab109507
Rabbit Anti XIAP	Abcam	Cat: ab229050
Rabbit Anti FAK (phospho Y397) antibody	Abcam	Cat: ab81298
Mouse Anti Talin 1	Abcam	Cat: ab108480
Rabbit Anti Talin 2	Abcam	Cat: ab108967
Rabbit Anti Ki-67	Abcam	Cat: ab16667
Rabbit Anti Anti-Hsp90 beta	Abcam	Cat: ab203085
Rabbit Anti Na/K ATPase	Abcam	Cat: ab76020
Rabbit Anti HDAC2	Abcam	Cat: ab32117
Rabbit Anti Cullin1	Abcam	Cat: ab75817
VeriBlot	Abcam	Cat: ab131366
Rabbit Anti β-Actin	Abclonal	Cat: AC026
Mouse Control IgG	Abclonal	Cat: AC011
Rabbit Control IgG	Abclonal	Cat: AC005
Purified Mouse IgG2a, κ Isotype Ctrl Antibody	BioLegend	Cat: 401501
Purified Rat IgG2b, κ Isotype Ctrl Antibody	BioLegend	Cat: 400643
Purified anti-mouse/human CD11b Antibody	BioLegend	Cat: 101248
Anti-human CD274 (B7-H1, PD-L1) Antibody	BioLegend	Cat: 329728
Purified Mouse IgG2b, κ Isotype Ctrl Antibody	BioLegend	Cat: 402201
Anti-mouse CD18 FITC	BioLegend	Cat: 101405
Anti-mouse CD11B FITC	BioLegend	Cat: 101205
Anti-human CD45 APC	BioLegend	Cat:304011
Anti-mouse CD45 APC	BioLegend	Cat:157605
Anti-human CD68 FITC	BioLegend	Cat:137005
Anti-human CD68 FITC	BioLegend	Cat:333805
Anti-human activated CD11B APC	BioLegend	Cat: 301409
PE anti-streptavidin antibody	BioLegend	Cat: 410503
Zombie Violet™ Fixable Viability Kit	BioLegend	Cat: 423113
Anti-mouse PD-L1 (B7-H1)	BIO x cell	Cat: BP0101
Rat IgG2b isotype control	BIO x cell	Cat: BP0090
Anti-mouse/human/rat CD47	BIO x cell	Cat: BE0283
Mouse IgG1 isotype control	BIO x cell	Cat: BE0083
Rabbit Anti SHP2	CST	Cat: 3397
Rabbit Anti Cleaved Caspase-3	CST	Cat: 9579
Rabbit Anti Phospho-Akt (Ser473)	CST	Cat: 4060

Rabbit Anti CD11b	CST	Cat: 17800
Rabbit Anti Histone H3	CST	Cat:4499
Rabbit Anti Vimentin	CST	Cat: 5741
Rabbit Anti Myosin Light Chain 2	CST	Cat: 3672
Rabbit Anti Cofilin	CST	Cat: 5175
Rabbit Anti Phospho-Cofilin (Ser3)	CST	Cat: 3311
Rabbit Anti Phospho-Paxillin (Tyr118)	CST	Cat: 69363
Rabbit Anti Phospho-Myosin Light Chain 2 (Thr18/Ser19)	CST	Cat: 95777
Rabbit Anti Phospho-p44/42 MAPK (Erk1/2) (Thr202/Tyr204)	CST	Cat: 4370
Mouse Anti Phospho-Tyrosine Alexa Fluor® 488 Conjugate)	CST	Cat: 9414
Rabbit Anti Phospho-Tyrosine	CST	Cat: 8954
Rabbit Anti Flag	CST	Cat: 14793
Rabbit AntI Phospho-SHP-1 (Tyr564)	CST	Cat: 8849
Mouse Anti HA	CST	Cat: 2367
Rabbit Anti INOS	CST	Cat:13120
Rabbit Anti INOS	CST	Cat:24595
Anti-Mouse CD80 APC	eBioscience	Cat: 17-0801-81
Anti-Mouse CD86 APC	eBioscience	Cat: 17-0862-81
Anti-Mouse MHCII PE	eBioscience	Cat: 12-5322-81
FDbio-Femto Ecl	FUDE biological	Cat: FD8030
Goat anti Mouse IgG (H+L) Alexa Fluor Plus 488	Invitrogen	Cat: A32723
Goat anti Rabbit IgG (H+L) Alexa Fluor Plus 488	Invitrogen	Cat: A32731
Goat anti Mouse IgG (H+L) Alexa Fluor Plus 555	Invitrogen	Cat: A32727
Goat anti Rabbit IgG (H+L) Alexa Fluor Plus 555	Invitrogen	Cat: A32732
Goat anti Rabbit IgG (H+L) Alexa Fluor Plus 647	Invitrogen	Cat: A32733
Goat anti Mouse IgG (H+L) Alexa Fluor Plus 647	Invitrogen	Cat: A32728
Donkey anti Goat IgG (H+L) Alexa Fluor Plus 647	Invitrogen	Cat: A32849
Rabbit Anti Kindlin 3	Invitrogen	Cat: PA5-116402
Rabbit Anti SENP8	Invitrogen	Cat: PA5-118500
Donkey Anti SHP2	Invitrogen	Cat: PA5-17956
Goat anti mouse IgG HRP	MultiSciences	Cat: 70-GAM007
Goat anti rabbit IgG HRP	MultiSciences	Cat: 70-GAR007
Rabbit anti Myc	Origene	Cat: TA591009

Mouse anti Myc	Origene	Cat: TA150121
Mouse anti-Flag	Origene	Cat: TA50011-100
HRP Goat Anti-Mouse IgG LCS	Abbkine	Cat: A25012
HRP Goat Anti-Mouse IgG HCS	Abbkine	Cat: A25112
Mouse Anti CD18	Santa Cruz	Cat: sc-8420
Mouse Anti CD68	Santa Cruz	Cat: sc-20060
Mouse Anti Paxillin	Santa Cruz	Cat: sc-365379
Mouse Anti SENP8	Santa Cruz	Cat: sc-271498
Mouse Anti Cullin5	Santa Cruz	Cat: sc-373822
Mouse Anti SHP1	Santa Cruz	Cat: sc-7289
Mouse Anti SHP2	Santa Cruz	Cat: sc-7384
Rabbit Anti UBE2F	Santa Cruz	Cat: sc-398668
Anti-streptavidin antibody	Santa Cruz	Cat: sc-52234
Duolink ® In Situ Red Starter Kit Mouse/Rabbit	Sigma	Cat: DUO92101
Mouse Anti NEDD8	Sigma	Cat: N2786
**Transfection Reagents**		
Mouse macrophage nucleofector Kit	Lonza	Cat: VVPA-1009
INTERFERin	Polyplus	Cat: 101000028
Lipo3000	Thermo	Cat: L3000008
**Other Reagents**		
Multi-fluorescent Immunohistochemical Staining Kit	Absin	Cat: abs50029
Biotinylated Mouse CD47 Protein	Acro biosystem	Cat: CD7-M82E4
SHP2 biosensor	Addgene	Cat: 134346
Hoechst 33342	Beyotime	Cat: C1028
JC-1	Beyotime	Cat: C2005
SureBeads™ Protein G Magnetic Beads	BIO-RAD	Cat:1614023
SureBeads™ Protein A Magnetic Beads	BIO-RAD	Cat:1614013
Biotin and Cy5 label of SHP2 Activating Peptide	BIOSS antibodies	N/A
pep-20-D12	Genesript	N/A
2P-IRS-1	Genesript	N/A
FBS	Gibco	Cat: 10099141C

DMEM	Gibco	Cat: 11995065
RPMI1640	Gibco	Cat: 61870036
0.25% Trypsin-EDTA	Gibco	Cat: 25200072
Advanced DMEM/F-12	Gibco	Cat: 12634010
Opti-MEM	Gibco	Cat: 51985034
HEPES	Gibco	Cat: 15630106
Glutamax	Gibco	Cat: 35050061
B-27 Supplement Minus Vitamin A	Gibco	Cat: 12587010
Penicillin-Streptomycin	Gibco	Cat: 15140122
PBS	Hyclone	Cat: SH30256.01
EDTA	Invitrogen	Cat: AM9260G
CFSE	Invitrogen	Cat: 65-0850-84
LysoTracker	Invitrogen	Cat: L7528
DiFMUP	Invitrogen	Cat: D6567
Streptavidin	Invitrogen	Cat: S888
CellMask Plasma Membrane Stains	Invitrogen	Cat: C10046
Wheat Germ Agglutinin Conjugates	Invitrogen	Cat: W32466
Dynabeads Sheep anti-Rat IgG	Invitrogen	Cat: 11035
Cell Proliferation Dye eFluor™ 450	Invitrogen	Cat: 65-0842-85
Cell Proliferation Dye eFluor™ 670	Invitrogen	Cat: 65-0840-85
Dynabeads Streptavidin Trial Kit	Invitrogen	Cat: 65801D
IL-6 Mouse Uncoated ELISA Kit	Invitrogen	Cat: 88-7064-88
IL-1 beta Mouse Uncoated ELISA Kit	Invitrogen	Cat: 88-7013-88
TNF alpha Mouse Uncoated ELISA Kit	Invitrogen	Cat: 88-7324-88
MCP-1/CCL2 Mouse Uncoated ELISA Kit	Invitrogen	Cat: 88-7391-88
IFN gamma Mouse Uncoated ELISA Kit	Invitrogen	Cat: 88-7314-88
Mouse interleukin 8 ELISA Kit	MyBioSource	Cat: MBS1601073
M-CSF	Novoprotein	Cat: CJ46
NovoRec® plus One-step PCR Cloning Kit	Novoprotein	Cat: NR005-01A
IL-2	Novoprotein	Cat: C013
hEGF	Novoprotein	Cat: C029

NEDD8 Conjugation Initiation Kit	R&D Systems	Cat: K-800
RhNEDD8 AMC	R&D Systems	Cat: UL-552-050
Protein A/G PLUS-Agarose	Santa Cruz	Cat: sc2003
MLN4924	Selleck	Cat: S7109
SHP099	Selleck	Cat: S6388
TPI-1	Selleck	Cat: S6570
TNO155	Selleck	Cat: S8987
Anti-FLAG®M2 Magnetic Beads	Sigma	Cat: M8823
3dGRO™ L-WRN Conditioned Media Supplement	Sigma	Cat: SCM105
PMA	Sigma	Cat: P1585
DNase I	Sigma	Cat: DN25
Nicotinamide	Sigma	Cat: 72340
N-Acetylcysteine	Sigma	Cat: A0737
SB-202190	Sigma	Cat: S7067
Human CEACAM5 ELISA kit	Solarbio	Cat: SEKH-0130
Streptavidin polystyrene particles	Spherotech	Cat: 73-SVP-50-5
DTBP	Thermo	Cat: 20665
BCA Protein Assay Kit	Thermo	Cat: 23225
Ham's F-12 Nutrient Mix powder	Thermo	Cat: 21700075
Pierce Glutathione Agarose Resin	Thermo	Cat: 16109
DAPI	Thermo	Cat: 62248
Subcellular Protein Fractionation Kit for Cultured Cells	Thermo	Cat: 78840
A83-01	Tocris	Cat: 2939
Gastrin	Tocris	Cat: 3006
Type I collagen gel	Trevigen	Cat: 3440-100-01
Collagenase IV	Worthington	Cat: LS004188
TRITC Phalloidin	Yeasen Biotechnology	Cat: 40734ES75
FITC Phalloidin	Yeasen Biotechnology	Cat: 40735ES75
**Plasmids**		
pLKO.1-TRC Cloning Vector	Addgene	Cat: 632561
psPAX2	Addgene	Cat: 12260
pMD2.G	Addgene	Cat: 12259
LentiCRISPRv2	Addgene	Cat: 10878
PXJ40(Myc, Flag, HA, mCherry)		N/A
**Squence for gene knockdown**		
siScramble: 5'-*UUCUCCGAACGUGUCACGUTT*-3'	General Biol Inc	N/A

siSENP8#1: 5'-*CCACUGGAGUUUAUUGGUCUA*-3'	General Biol Inc	N/A
siSENP8#2:5'-*GCAUACAUCACAAAGAAGATT*-3',	General Biol Inc	N/A
siSENP8#*3:5'-ACCAACUUAUUUGAACAUUUA-3'*	General Biol Inc	N/A
ShScramble*: 5'-CCTAAGGTTAAGTCGCCCTCG-3',*	TsingkeBiotechnology	N/A
ShSHP2*: 5'-TTGAGACCAAGTGCAACAATT-3'*	TsingkeBiotechnology	N/A
ShSIRPα1： 5'-*AAGTGAAGGTGACTCAGCCTG*-3'		
ShSIRPα2： 5'-*AATCAGTGTCTGTTGCTGCTG*-3'		
gRNA hSENP8*#1: 5'-CCATGTAACTCAAGACTACG-3'*	TsingkeBiotechnology	N/A
gRNA hSENP8*#2: 5'-CAACTCAGTTCACGCAAAGC-3'*	TsingkeBiotechnology	N/A
**Primers for mice identification**		
Lysm^{cre}-M-F*: 5'-CCCAGAAATGCCAGATTACG-3'*	TsingkeBiotechnology	N/A
Lysm^{cre}-M-R*: 5'-CTTGGGCTGCCAGAATTTCTC-3'*	TsingkeBiotechnology	N/A
Lysm^{cre}-WT-F*: 5'-TTACAGTCGGCCAGGCTGAC-3'*	TsingkeBiotechnology	N/A
Ube2m$^{fl/fl}$*-F: 5'-CCGTGTCGTGAAGATTGTGAAGG-3'*	TsingkeBiotechnology	N/A
Ube2m$^{fl/fl}$*-R: 5'-ACCTCCACTGTCCTTCCTCGTCTC-3'*	TsingkeBiotechnology	N/A
Ube2f $^{fl/fl}$*-F: 5'-CCAGGGTGGAAAATTTCAGTTT-3'*	TsingkeBiotechnology	N/A
Ube2f $^{fl/fl}$*-R1: 5'-GCGAGCTCAGACCATAACTTCG-3'*	TsingkeBiotechnology	N/A
Ube2f $^{fl/fl}$*-R2: 5'-CCCTGGAATTTCGGTATTATA-3'*	TsingkeBiotechnology	N/A
Shp2$^{fl/fl}$*-F: 5'-CAGTTGCAACTTTCTTACCTC-3'*	TsingkeBiotechnology	N/A
Shp2$^{fl/fl}$*-R: 5'-GCAGGAGACTGCAGCTCAGTGATG-3'*	TsingkeBiotechnology	N/A
Senp8-F: 5'-CAGGAGACAGAGGCAGAAGAA-3'	TsingkeBiotechnology	N/A
Senp8-R1: 5'-GGATATTGTACTCACATGACCAAGA-3'	TsingkeBiotechnology	N/A
Senp8-R2: 5'-GGATTAAGTTTGAGGAAGGTGACA-3'	TsingkeBiotechnology	N/A
Senp8$^{fl/fl}$*-F: 5'-CCAGGAACACTGATTCCTATGCAC-3'*	TsingkeBiotechnology	N/A
Senp8$^{fl/fl}$*-R: 5'-AATGGATGTGACAGTGGTGAGAG-3'*	TsingkeBiotechnology	N/A
**Primers for QPCR**		
Human *SENP8* F:*5'-ACTGCGGCAATCAGATGTCTC-3'*	TsingkeBiotechnology	N/A
Human *SENP8* R:*5'-GGAACATGGCAATCTCTGCTG-3'*	TsingkeBiotechnology	N/A
Human *UBA3* F:*5'-AAGCACTTCACTACGCTTAGC-3'*	TsingkeBiotechnology	N/A
Human *UBA3* R:*5'-TGGAGTATGACTGTGGTCCTTT-3'*	TsingkeBiotechnology	N/A
Human *UBE2M* F:*5'-ATGAGGGCTTCTACAAGAGTGG-3'*	TsingkeBiotechnology	N/A
Human *UBE2M* R:*5'-ATTGTCTCACACTTCACCTTGG-3'*	TsingkeBiotechnology	N/A
Human *XIAP* F:*5'-ACCGTGCGGTGCTTTAGTT-3'*	TsingkeBiotechnology	N/A
Human *XIAP* R:*5'-TGCGTGGCACTATTTTCAAGATA-3'*	TsingkeBiotechnology	N/A
Human *18s* rRNA F*:5'-GTAACCCGTTGAACCCCATT-3'*	TsingkeBiotechnology	N/A
Human *18s* rRNA R*:5'-CCATCCAATCGGTAGTAGCG-3'*	TsingkeBiotechnology	N/A

Supplemental Table 2

Gender	IHC	Tumor location	Gross appearance	Histology
Male	MSS	Sigmoid colon	Ulcerative type	Moderately differentiated adenocarcinoma
Male	MSS	Rectum	Ulcerative type	Moderately differentiated adenocarcinoma
Male	MSS	Sigmoid colon	Ulcerative type	Moderately differentiated adenocarcinoma
Male	MSS	Left-sided colon-Rectum	Ulcerative type	Moderately differentiated adenocarcinoma
Female	MSS	Left-sided colon	protruded type	Mucinous carcinoma
Female	MSS	Left-sided colon	ulcerative-infiltrating type	Moderately differentiated adenocarcinoma
Male	MSS	Sigmoid colon	protruded type	Moderately differentiated adenocarcinoma
Female	MSS	Sigmoid colon	ulcerative type	Moderately differentiated adenocarcinoma
Female	MSS	Right-sided colon	ulcerative type	Moderately differentiated adenocarcinoma
Male	MSS	Right-sided colon	ulcerative type	Moderately differentiated adenocarcinoma
Male	MSS	Rectum	ulcerative type	Moderately differentiated adenocarcinoma
Male	MSS	Right-sided colon	ulcerative type	Moderately-Poorly differentiated adenocarcinoma
Female	MSS	Sigmoid colon	ulcerative type	Moderately differentiated adenocarcinoma
Female	MSS	Rectum	ulcerative type	Moderately differentiated adenocarcinoma
Male	MSS	Rectum	ulcerative type	Moderately differentiated adenocarcinoma
Male	MSS	Sigmoid colon	ulcerative type	Moderately differentiated adenocarcinoma

Gender	IHC	Tumor location	Gross appearance	Histology
Female	MSS	Right-sided colon	ulcerative type	Moderately differentiated adenocarcinoma
Male	MSS	Sigmoid colon	ulcerative type	Moderately differentiated adenocarcinoma
Female	MSS	Right-sided colon	ulcerative type	Moderately-poorly differentiated adenocarcinoma
Female	MSS	Sigmoid colon	ulcerative type	Well-moderately differentiated adenocarcinoma
Female	MSS	Rectum	ulcerative type	Moderately differentiated adenocarcinoma
Male	MSS	Rectum	ulcerative type	Signet ring cell carcinoma
Male	MSS	Rectum	ulcerative type	Poorly differentiated adenocarcinoma
Male	MSS	Right-sided colon	infiltrating type	Moderately differentiated adenocarcinoma
Female	MSS	Right-sided colon	protruded type	Moderately differentiated adenocarcinoma
Male	MSS	Rectum	protruded type	Moderately-poorly differentiated adenocarcinoma
Female	MSS	Left-sided colon	protruded type	Moderately differentiated adenocarcinoma
Male	MSS	Left-sided colon	ulcerative type	Moderately differentiated adenocarcinoma
Male	MSS	Left-sided colon	infiltrating type	Moderately-poorly differentiated adenocarcinoma
Female	MSS	Right-sided colon	ulcerative type	Moderately-poorly differentiated adenocarcinoma
Male	MSS	Rectum	ulcerative type	Moderately differentiated adenocarcinoma
Male	MSS	Left-sided colon	ulcerative type	Moderately-poorly differentiated adenocarcinoma
Male	MSS	Rectum	protruded type	Moderately differentiated adenocarcinoma
Male	MSS	Rectum	ulcerative type	Well-moderately differentiated adenocarcinoma
Male	MSS	Sigmoid colon	ulcerative type	Well-moderately differentiated adenocarcinoma
Female	MSS	Rectum	ulcerative type	Moderately-poorly differentiated adenocarcinoma
Female	MSS	Sigmoid colon	ulcerative type	Moderately differentiated adenocarcinoma
Male	MSS	Rectum	ulcerative type	Mucinous carcinoma

Male	MSS	Sigmoid colon	ulcerative type	Moderately differentiated adenocarcinoma
Female	MSS	Right-sided colon	ulcerative type	Moderately differentiated adenocarcinoma
Male	MSS	Right-sided colon	ulcerative type	Well-moderately differentiated adenocarcinoma
Female	MSS	Rectum	protruded type	Well-moderately differentiated adenocarcinoma
Female	MSS	Rectum	ulcerative type	Moderately-poorly differentiated adenocarcinoma
Male	MSS	Right-sided colon	ulcerative type	Moderately differentiated adenocarcinoma
Male	MSS	Right-sided colon	ulcerative type	Moderately differentiated adenocarcinoma
Male	MSS	Rectum	ulcerative type	
Male	MSS	Rectum	ulcerative type	Moderately differentiated adenocarcinoma
Male	MSS	Rectum	ulcerative type	Well-moderately differentiated adenocarcinoma
Male	MSS	Rectum	protruded type	Well-moderately differentiated adenocarcinoma
Male	MSI	Right-sided colon	ulcerative type	Poorly differentiated adenocarcinoma
Male	MSI	Rectum	protruded type	Moderately differentiated adenocarcinoma
Male	MSI	Left-sided colon	ulcerative type	Moderately differentiated adenocarcinoma
Female	MSI	Right-sided colon	ulcerative type	Well-moderately differentiated adenocarcinoma
Male	MSI	Right-sided colon	protruded type	Moderately-poorly differentiated adenocarcinoma
Male	MSI	Right-sided colon	ulcerative type	Well-moderately differentiated adenocarcinoma
Female	MSI	Sigmoid colon	protruded type	Moderately differentiated adenocarcinoma
Female	MSI	Right-sided colon	ulcerative type	Well-moderately differentiated adenocarcinoma
Male	MSI	Right-sided colon	ulcerative type	Well-moderately differentiated adenocarcinoma
Male	MSI	Right-sided colon	protruded type	Moderately differentiated adenocarcinoma
Male	MSI	Right-sided colon	protruded type	Moderately-poorly differentiated adenocarcinoma

Female	MSI	Right-sided colon	protruded type	Moderately differentiated adenocarcinoma
Male	MSI	Rectum	protruded type	Well-moderately differentiated adenocarcinoma
Male	MSI	Right-sided colon	protruded type	Well-moderately differentiated adenocarcinoma
Male	MSI	Right-sided colon	ulcerative type	Well-moderately differentiated adenocarcinoma
Female	MSI	Sigmoid colon	protruded type	Well differentiated adenocarcinoma
Male	MSI	Rectum	ulcerative type	Moderately differentiated adenocarcinoma
Male	MSI	Rectum	ulcerative type	Moderately differentiated adenocarcinoma
Female	MSI	Right-sided colon	ulcerative type	Moderately differentiated adenocarcinoma
Male	MSI	Right-sided colon	protruded type	Moderately differentiated adenocarcinoma
Male	MSI	Right-sided colon	ulcerative type	Poorly differentiated adenocarcinoma
Female	MSI	Rectum	ulcerative type	Moderately differentiated adenocarcinoma
Male	MSI	Right-sided colon	protruded type	Moderately-poorly differentiated adenocarcinoma
Male	MSI	Transverse colon	ulcerative type	Moderately-poorly differentiated adenocarcinoma
Male	MSI	Right-sided colon	protruded type	Moderately differentiated adenocarcinoma
Male	MSI	Right-sided colon	ulcerative type	Poorly differentiated adenocarcinoma
Male	MSI	Right-sided colon	protruded type	Well-moderately differentiated adenocarcinoma
Male	MSI	Right-sided colon	protruded type	Moderately-poorly differentiated adenocarcinoma
Male	MSI	Right-sided colon	ulcerative type	Moderately-poorly differentiated adenocarcinoma
Male	MSI	Colon	ulcerative type	Moderately-poorly differentiated adenocarcinoma
Female	MSI	Left-sided colon	protruded type	Well-moderately differentiated adenocarcinoma

Supplemental Table 3

Gender	Patient	Histology
Male	Patient1	Poorly differentiated sarcomatoid carcinoma
Male	Patient2	Moderately differentiated squamous cell carcinoma
Male	Patient3	Moderately differentiated invasive adenocarcinomas (acinar and lepidic)
Female	Patient4	Nonmucinous adenocarcinoma in situ
Female	Patient5	Moderately differentiated invasive adenocarcinomas (acinar, lepidic and papillary)
Female	Patient6	Moderately differentiated invasive adenocarcinomas (acinar)
Male	Patient7	Poorly differentiated invasive adenocarcinomas (micropapillary and solid)

Supplemental Table 4

1	89Y	CD45	22	160Gd	CD86
2	115In	CD3e	23	161Dy	iNOS
3	141Pr	CD103	24	162Dy	CD206
4	142Nd	MHCII	25	163Dy	CD25
5	143Nd	B220	26	164Dy	CD40
6	144Nd	CX3CR1	27	165Ho	Ly6G
7	145Nd	CD69	28	166Er	NEDD8
8	146Nd	PD1	29	167Er	CD16/32
9	147Sm	CD80	30	168Er	FoxP3
10	148Nd	Ly6C	31	169Tm	SENP8
11	149Sm	CD64	32	170Er	PDL1
12	150Nd	CD14	33	171Yb	CD163
13	151Eu	NK1.1	34	172Yb	CD127
14	152Sm	CD11c	35	173Yb	CD172a
15	153Eu	TCRgd	36	174Yb	CCR2
16	154Sm	Ki-67	37	175Lu	Siglec F
17	155Gd	BST2	38	176Yb	MERTK
18	156Gd	CD68	39	197Au	CD4
19	157Gd	FceRIa	40	198Pt	CD8a
20	158Gd	CD19	41	209Bi	CD11b
21	159Tb	F4/80		191/193Ir	DNA
				194Pt	Live/dead
